# Parent–adolescent interaction quality and adolescent affect—An experience sampling study on effect heterogeneity

**DOI:** 10.1111/cdev.13733

**Published:** 2022-01-31

**Authors:** Anne Bülow, Eeske van Roekel, Savannah Boele, Jaap J. A. Denissen, Loes Keijsers

**Affiliations:** ^1^ 6984 Department of Psychology, Education & Child Studies Erasmus University Rotterdam Rotterdam The Netherlands; ^2^ 7899 Department of Developmental Psychology Tilburg University Tilburg The Netherlands; ^3^ 8125 Department of Developmental Psychology Utrecht University Utrecht The Netherlands

## Abstract

Person–environment interactions might ultimately drive longer term development. This experience sampling study (Data collection: 2019/20 the Netherlands) assessed short‐term linkages between parent–adolescent interaction quality and affect during 2281 interactions of 124 adolescents (*M*_age_ = 15.80, *SD*_age _= 1.69, 59% girls, 92% Dutch, Education: 25% low, 31% middle, 35% high, 9% other). Adolescents reported on parent–adolescent interaction quality (i.e., warmth and conflict) and momentary positive and negative affect five to six times a day, for 14 days. Preregistered dynamic structural equation models (DSEM) revealed within‐family associations between parent–adolescent interaction quality and adolescent affect (concurrently: *r *= −.22 to .39; lagged effects: *ß* = −.17 to .15). These effects varied significantly between families. These findings stress the need for more person‐specific research on parenting processes.

**Abbreviations**
DSEM: dynamic structural equation model
DST: dynamic systems theory
ESM: experience sampling method
ML‐AR: multilevel autoregressive model
ML‐VAR: multilevel vector autoregressive model

Being a parent entails the unique opportunity (and challenge) to support children in their maturation. Especially in adolescence, where children's well‐being is under pressure (Solmi et al., 2021), parents’ support can be much needed. In ecological models of development, parent–child relationships are considered one of the most proximal and important influences upon child development (Bronfenbrenner, 1979; Sameroff, 2010). Decades of research have indeed shown that parent–adolescent relationships that are characterized by warmth (i.e., provision of affection, love, and support) and few conflicts (i.e., quarreling and disagreements) are associated with adolescent well‐being (for meta‐analytical evidence see: Khaleque, 2013; Weymouth et al., 2016). In this study, we focus on one component of well‐being, namely affective wellbeing, which is defined as experience of positive and negative emotions in daily life (i.e., affect; Ben‐Arieh et al., 2014; Ebesutani et al., 2012). How these longer term effects of parenting upon adolescent affect come about in daily life, however, is yet to be understood.

## Micro process of parenting

According to dynamic systems theory (DST; Kunnen, 2018; De Ruiter et al., 2019; Smith & Thelen, 2003), development is a process that emerges from dynamic reciprocal exchanges between a child and his or her environment. Each interaction that parents have with their adolescent child may carry over to the next and feed back into the system, gradually carving out their family‐specific developmental pathway of their relationship and their affective well‐being (e.g., De Ruiter et al., 2019). Hence, longer term processes spanning years emerge from the repeated bidirectional associations between parent–adolescent interactions and subsequent adolescent affective well‐being. This dynamic view on how parenting and adolescent affective well‐being are intertwined stresses three points: (1) the importance to study parenting processes on a *within*‐*family level*, (2) at the *micro timescale*, and (3) accounting for *family*‐*specific processes*.

Theoretical considerations and empirical evidence indicate that aggregated between‐family associations (i.e., differences between families) do not contain information about the underlying *within*‐*family processes* (i.e., individual change over time; Dietvorst et al., 2018; Keijsers & Van Roekel, 2018). It is an open question, moreover, whether evidence from macro timescales (i.e., long‐term associations over years) can be applied to draw inferences regarding the *micro processes*, which take place within the individual family. Therefore, in order to understand the smallest building blocks of development, there is a call for more studies that specifically zoom in on micro processes (Bamberger, 2016; Boele et al., 2020; Dormann & Griffin, 2015; Keijsers & Van Roekel, 2018; Larson, 2019). Finally, one of the pressing questions in the parenting literature is the extent to which parenting processes are universal or *family*‐*specific*. Ecological theories of development (Bronfenbrenner, 1979) and differential susceptibility theory (Pluess & Belsky, 2010) consider that each individual is unique and may react differently to parent–adolescent interactions (i.e., effect heterogeneity).

Despite the increasing popularity of DST for understanding the complexity of adolescent's development, empirical insights into the fine‐grained building blocks of development are still scarce. Existing evidence is mostly based on comparing families with each other at macro timescales and is not accounting for differences between families (for a review see: Boele et al., 2020). This leaves important lacunas in our knowledge. This preregistered study is the first to unravel micro processes that link momentary parent–adolescent interaction quality (in terms of warmth and conflict) to subsequent affective well‐being (in terms of positive and negative affect), and vice versa, at the within‐family level. Moreover, it assesses how families differ in these processes.

## Methods to study micro processes

To assess these micro processes in parenting there are two prominent approaches. One approach is to use *observational studies*, in which videotaped parent–adolescent interactions are analyzed, which provides insights into observable behaviors within a single interaction. Such studies showed that interaction quality and affect are linked on the interaction level. Adolescents expressed more negative emotions in a conflict interaction than in discussions about a positive topic (e.g., Hollenstein & Lewis, 2006). The other approach to measure short‐term effects of parenting is to use *daily diary studies*. In these studies, participants fill out questionnaires one time a day, typically at the end of the day. This method can capture fluctuations in daily parent–adolescent relationship quality in real life. Such studies for example demonstrated that on days when adolescents reported more parental warmth and less parent–adolescent conflict, they felt more positive and less negative affect (e.g., Bai et al., 2017; Flook, 2011; Janssen et al., 2020; Robles et al., 2016). However, findings are inconsistent whether parent–adolescent conflict and parental warmth can also predict next day's affect (e.g., Boele et al., 2021; LoBraico et al., 2019; Timmons & Margolin, 2015). Some studies showed substantial effect heterogeneity between families in how interaction quality and adolescent affect were linked (Borghuis et al., 2020; Chung et al., 2009, 2011; Janssen et al., 2020; Timmons & Margolin, 2015). For instance, whereas some adolescents may respond quite strongly to parent–adolescent interactions, others may be unaffected (e.g., Janssen et al., 2020). Individual factors (i.e., gender, depressive symptoms) were associated with these differences (Chung et al., 2009; Janssen et al., 2020; Timmons & Margolin, 2015).

Extending this empirical body of work, the current study focuses on the processes that take place within days in individual families. It thus bridges the empirical gap in our knowledge regarding the intermediate timescale between existing observational studies assessing single interactions (seconds or minutes) and daily diary studies assessing the daily experienced relationship quality (1 day). That is, we measured fluctuations in parent–adolescent interactions quality across the day with the experience sampling method (ESM, also called “ecological momentary assessment,” EMA). In ESM, participants answer multiple questionnaires per day on their smartphone (Bolger et al., 2003; Larson, 2019; Repetti et al., 2015). The questionnaires can capture information about real‐life situations or dynamic states such as the current affect or the experienced quality of parent–adolescent interactions.

Compared to daily diary studies, which tap into the quality of all interactions over 1 day, ESM and observational studies give a better proxy of this smallest dyadic unit: the parent–adolescent interaction. Zooming in on interactions may help discover short‐lived parenting processes. For example, if effects of a parenting interaction on affect lingers for only several hours, daily diary studies could not detect it at the next day's measurement, which could explain inconsistent cross‐lagged findings in daily diary studies (Boele et al., 2021; LoBraico et al., 2019; Timmons & Margolin, 2015). Compared to observational studies, which mostly investigate only one interaction (e.g., Crowell et al., 2017), ESM is suited to measure patterning of interactions over time, which is essential for describing and understanding how relationships are built (Hinde, 1976).

Despite ESM is a feasible and increasingly popular method in adolescent samples (Larson, 2019; Van Roekel et al., 2019), only a handful of studies have employed ESM to assess how parent–adolescent interactions are intertwined with adolescent's affect in daily life. These studies, for instance, demonstrated that on moments when parents were present, adolescents felt better than when they were alone (Offer, 2013; Schneiders et al., 2007). However, they felt even better on moments when they were with friends (Kim et al., 2018; Larson, 1983). Even though there are a few studies measuring the *presence* of family with ESM (e.g., Offer, 2013), we know of no ESM study that investigated how the *quality* of earlier parent–adolescent interactions may affect adolescent affect, or vice versa. Previous studies on adult samples, however, suggest that the quality of the interaction might be more strongly associated with affect than the presence of others (Liu et al., 2019).

## The current study

DST (Smith & Thelen, 2003) stresses the importance of understanding the sequences of parent–adolescent interactions in daily life as building blocks of relationships and adolescent development. This study aimed to unravel how warmth and conflict in parent–adolescent interactions with primary caregivers can predict later adolescent affect and vice versa within a day, accounting for possible individual differences in these effects. As the perception of parent–adolescent interaction is theoretically thought to be predominantly determining its effects on adolescent affective well‐being (Soenens et al., 2015), this study focuses on adolescents’ reports. We preregistered the following hypotheses for this ESM study (see https://osf.io/v6g2m/):

H1. Parenting interactions and adolescent's affect are linked within families.

H1.1. Parental warmth is associated with more positive affect and less negative affect at the current moment as well as at the next moment.

H1.2. Parent–adolescent conflict is associated with more negative affect and less positive affect at the current as well as at the next moment.

H2. Parenting interactions and adolescent's affect are associated between families.

H2.1. Higher levels of warmth are associated with higher levels of positive affect and lower levels of negative affect.

H2.2. Higher levels of parent–adolescent conflict are associated with lower levels of positive affect and higher levels of negative affect.

H3. The within‐family associations described under H1 differ between families (i.e., effect heterogeneity).

## METHOD

### Sample

One hundred and twenty‐four participants were included in the current study — of the 129 participants that took part in the study, five were excluded from the analysis (*n *= 3 no interactions with parents, *n *= 2 careless responding). Participants were on average 15.80 years old (*SD*_age _= 1.69, Range_age_ = 12–18). Fifty‐nine percent were girls, and the majority (92%) had the Dutch nationality (5% did not indicate their nationality, 3% came from Asian and African countries). In 15% of participants, at least one parent was not born in the Netherlands (5% Asia, 4% Europe, 2% Africa, 2% Caribbean). The adolescents followed different educational tracks: Twenty‐five percent followed a lower educational track (pre‐vocational secondary education and vocational training), 31% followed a medium educational track (higher general secondary education or university of applied sciences), 35% followed a higher educational track (pre‐university secondary education or university), and 9% could not be assigned to an educational track (*n* = 7 no report, *n* = 3 do not go to school, *n* = 1 elementary education, *n* = 1 mixed‐track). Parents had a diverse educational background: 3% did not have a secondary education, 26% had vocational training, 33% graduated from a university of applied sciences, and 20% graduated from university (13% of adolescents did not know what education they parents had, and 5% of adolescents did not indicate an education level of their parents).

Most participants (*N* = 118, 95% of sample) reported on their family situation. Of these participants, most participants indicated to live together with their mother and father in one home (75%), a minority lived together with their mother and father in separate homes (14%) or lived only with their mother (10%). One participant indicated to not live with their parents but with their legal guardian. Most participants reported to have contact with their mother (97%) and father (89%), while some participants also reported contact with their stepmother (7%) or stepfather (12%). Furthermore, some participants reported contact to other parental figures, like their adoption mother (*n *= 1), adoption father (*n *= 1), legal guardian (*n *= 1), or second mother (*n *= 1).

The sample was composed of two substudies with identical instruments and could therefore be combined. Most participants took part in the DESPAI study (Codebook: https://osf.io/vstrn), where *N *= 99 adolescents (56% girls, *M*_age _= 15.81, *SD*_age _= 1.72) participated in 2020 (Feb 3–16, before first COVID‐19 patient in the Netherlands). Additionally, *N* = 30 adolescents (70% girls, *M*_age _= 15.83, *SD*_age _= 1.51) took part in the pilot study in 2019 (March 25–April 7), with the same instruments and a comparable sampling scheme (deviations are reported in the Appendix).

### Procedure

The convenience sample was recruited by 41 Dutch undergraduate psychology students, which informed acquainted adolescents about the study. The inclusion criteria were as follows: adolescent participants aged between 12 and 18 years, no diagnosis of any psychiatric, developmental, or substance use disorders, and no substantial visual or hearing impairments. Furthermore, only one adolescent per nuclear family could participate (i.e., no siblings). This study was approved by the Ethical Committee of Tilburg University (EC‐2017.105a). We follow the guidelines from Van Roekel and colleagues (2019) for reporting an ESM study.

During a home visit, participants were informed about the study, had the possibility to ask questions and got help with installing the Ethica Data app (Ethica Data Sevices Inc, 2019) on their smartphone. Both Android (41%) and iOS (59%) operating systems were used. Upon participants’ and their parents’ active informed consent (parents only gave active informed consent for adolescents under the age of 16), participants were sent an online questionnaire provided through Qualtrics, to assess demographics and information about parenting and well‐being at baseline (e.g., depressive symptoms). The next day the ESM period started. Most participants answered the questionnaire before the ESM period started (56%), some did it during the ESM period (40%) and a minority did not fill in the baseline questionnaire (4%).

Participants could maximally earn 15 € in total (approx. $ 18). They received 5€ per week when they answered 75% of the ESM questionnaires and 3€ when they answered 65%–74% of the ESM questionnaires. Furthermore, they received 5€ for filling out the online questionnaires. Afterward, adolescents received a personal affect profile based on their ESM data and were invited to a guest lecture on adolescent development at the university.

### Sampling scheme and study design

The sampling scheme consisted of 14 days, with five measures per day from Monday until Friday and six measures per day during the weekend (*t*_max_ = 74). Participants were notified to fill out a questionnaire on their smartphones within 30 min after the initial notification (i.e., signal‐contingent sampling scheme). To increase compliance, participants received automatic reminders after 20 min, and all participants were regularly contacted via text messages informing them about their compliance rates. On average participants answered the questionnaires 8.21 min (*SD* = 7.87) after receiving the prompt.

Questionnaires were sent at semi‐random intervals. Participants received one questionnaire in the morning (07:00–07:30), two in the afternoon (15:30–16:10; 17:30–18:10) and two in the evening (19:30–20:10, 21:30–22:00). These were selected because interactions with parents were plausible during these time slots and to accommodate adolescents not having to fill it out during school hours. During the weekend, a different sampling scheme was employed: The morning questionnaire (07:00–07:30) was replaced with one questionnaire in the later morning (11:30–12:10) and one in the early afternoon (13:30–14:10), to adapt to adolescents getting up late and not attending school during the weekend.

Per questionnaire participants answered 25–37 items which took participants approx. 3–5 min to complete. When participants indicated that they had seen their parents in the last hour, they received follow‐up questions about the perceived interaction quality in terms of warmth and conflict. The reported interactions were on average 13.62 min ago (*SD* = 15.24; *Md* = 9; Range = 0–60). Participants received other items if they indicated that they had not seen their parents, to balance questionnaire length. This was done to prevent adolescents indicating that they have not seen their parents to avoid longer questionnaires. A slightly different scheme was used for the pilot study, see Appendix B. All items and a detailed description of the procedure can be found on OSF: https://osf.io/vstrn.

#### Compliance

One of the quality markers of ESM is compliance. In this study, of the 9060 planned questionnaires, 7530 surveys were delivered to the participants (83%). Technical errors that prevented surveys to be sent consisted of errors in programming surveys, incompatibilities with certain smartphones, and broken smartphones. From the delivered surveys, participants filled out 5301 questionnaires. Therefore, the participants compliance was 70% (Range = 23%–97%), which is typical in adolescent samples (Van Roekel et al., 2019). In personal communication with the research team, participants reported several reasons for noncompliance: being at work or school, studying, doing sports, being ill, sleeping late, or having to hand in their phone. In 3174 cases (60% of answered questionnaires), participants indicated that they had interacted with their parents. In 2281 cases (43% of answered questionnaires), this interaction was with their primary caregiver (*M* = 18.41 parent–adolescent interactions per participant, Range = 1–55). We defined the primary caregiver as the parent they reported the most interactions with in the ESM questionnaires. Most primary caregivers were mothers (*N *= 109, 88%), 14 were fathers (11%), and one was a legal guardian (<1%). These 2,281 interactions with the primary caregiver were included in the analysis (95% of preregistered sample size estimation, see power analysis).

### ESM measures

#### Momentary parent–child interaction

Because no ESM instrument existed for measuring perceived quality of parent–adolescent interactions, we developed and evaluated a novel instrument for this study based on existing items, literature, and input from adolescents. The items were sampled and reformulated for ESM from the Positive Parent Involvement Scale for Children (YES I AM Scale; Repetti, 1996; Robles et al., 2016), as well as two well‐established and for Dutch population validated instruments for assessing these parenting dimensions in surveys, namely the Level of Expressed Emotion Questionnaire (Cole & Kazarian, 1988) and the Network of Relationships Inventory (NRI; Furman & Buhrmester, 1985). We tested the content validity of these sets of items among 65 adolescents. During a lecture at a local high school, we asked them to report using mobile surveys what their parents were doing during positive and during negative interactions (“Think at moments with your parents that are (not) nice/(un‐)pleasant. What are your parents doing then?”). The content of their answers was highly overlapping with the coverage of the selected items. Based on high school students’ reports, we added one item, namely “My parent understood me” (warmth). Psychometric properties were subsequently evaluated in the pilot ESM study (*N* = 30; 70% girls, *M*_age _= 15.83, *SD*_age _= 1.51) of 13 days (see Supplemental Materials). Multilevel confirmatory factor analysis confirmed that the items of warmth and conflict were loading on one factor, respectively. Based on low within‐family and between‐family factor loadings, the item “My parent interfered with my life.” (conflict) was excluded.

The final version of the Momentary Parent–Child Interaction questionnaire (MPCI) consists of five items for parental warmth (e.g., “My parent and I got along well.”) and five items for parent–adolescent conflict (e.g., “My parent and I disagreed.”). Participants were presented with these items, if they indicated that they had seen and talked to one of their parents and/or caregivers in the past hour. Participants answered on a visual analogue scale (VAS) from 0 (strongly disagree) to 100 (strongly agree). We chose VAS scales, as they have similar psychometric properties as Likert scales, however, high‐school students prefer VAS scales above Likert scales (Tucker‐Seelley, 2008). Both scales showed excellent within‐family and between‐family reliability (warmth: *ω_w_* = .85, *ω_b_* = .97; conflict: *ω_w_* = .87, *ω_b_* = .95; Geldhof et al., 2014). All items are listed in Appendix A. The syntax and results of the multi‐level confirmatory factor analysis for the analytic sample are available in the Supplemental Materials.

#### Momentary positive and negative affect

Participants current positive and negative affect was measured with the shortened version of the Positive and Negative Affect Schedule for Children (PANAS‐C; Ebesutani et al., 2012). The validated questionnaire consisted of five items for positive affect (joyful, cheerful, happy, lively, proud) and five items for negative affect (miserable, mad, afraid, scared, sad). Participants rated their momentary affect on a VAS from 0 (strongly disagree) to 100 (strongly agree). Both scales have been used in other studies on adolescents (e.g., Schmidt et al., 2016), and in this study they showed moderate to excellent within‐ and between‐family reliability (PA: *ω_w_* = .85, *ω_b_* = .95; NA: *ω_w_* = .64, *ω_b_* = .84; Geldhof et al., 2014).

### Baseline measures

#### Depressive symptoms

Depressive symptoms were measured in the online questionnaire with the 10‐item version of the Reynolds Adolescent Depression Scale Second Edition (RADS‐2 Reynolds, 2005). Items were answered on a 4‐point Likert scale from 1 (almost never) to 4 (often). An example item is: “I am sad.” The scale had good between‐person reliability (*ω_b_* = .91).

### Power analysis

In an ESM study, power comes both from the number of individuals and the number of observations (Schultzberg & Muthén, 2018). Prior to data collection of the main study (after collection of the pilot study), a power analysis was conducted and preregistered (https://osf.io/v6g2m/) to determine the required sample size and number of observations, using Monte Carlo simulations in M*plus* (Version 8.3; Muthén & Muthén, 2019) and R (Hallquist & Wiley, 2018; R Core Team, 2019) (see Supplemental Materials). We plugged in estimates based on an earlier independent pilot study with 49 Dutch adolescents (collected in 2018 with 952 mother‐adolescent interactions). These indicated that we would need a sample of *N* = 120 with *t* = 20 (120 × 20 = 2400 observations) to estimate a small cross‐lagged effect of *β* = .08. Ultimately, our sample of *N* = 124 participants yielded an average of 18.41 interactions, and 2281 reported interactions in total (95% of preregistered sample size estimation).

### Preregistered analysis plan

Our analysis plan was preregistered before data collection of the main study (after collection of pilot data) and can be found on OSF (https://osf.io/v6g2m/). To investigate our hypotheses with sufficient statistical power, we used interactions of only one parent, as interaction dynamics might differ between parents and can therefore not be merged. We included the parent they reported the most interactions with during the ESM period (i.e., primary caregiver). We estimated several bivariate multilevel vector autoregressive models (ML‐VAR (1); Schuurman et al., 2016) using Dynamic Structural Equation Modelling (DSEM; McNeish & Hamaker, 2019) in M*plus* (Version 8.3, Muthén & Muthén, 2019; syntax in Supplemental Materials). Four models were estimated (2 [warmth/conflict] × 2 [positive affect/negative affect]).

**FIGURE 1:**
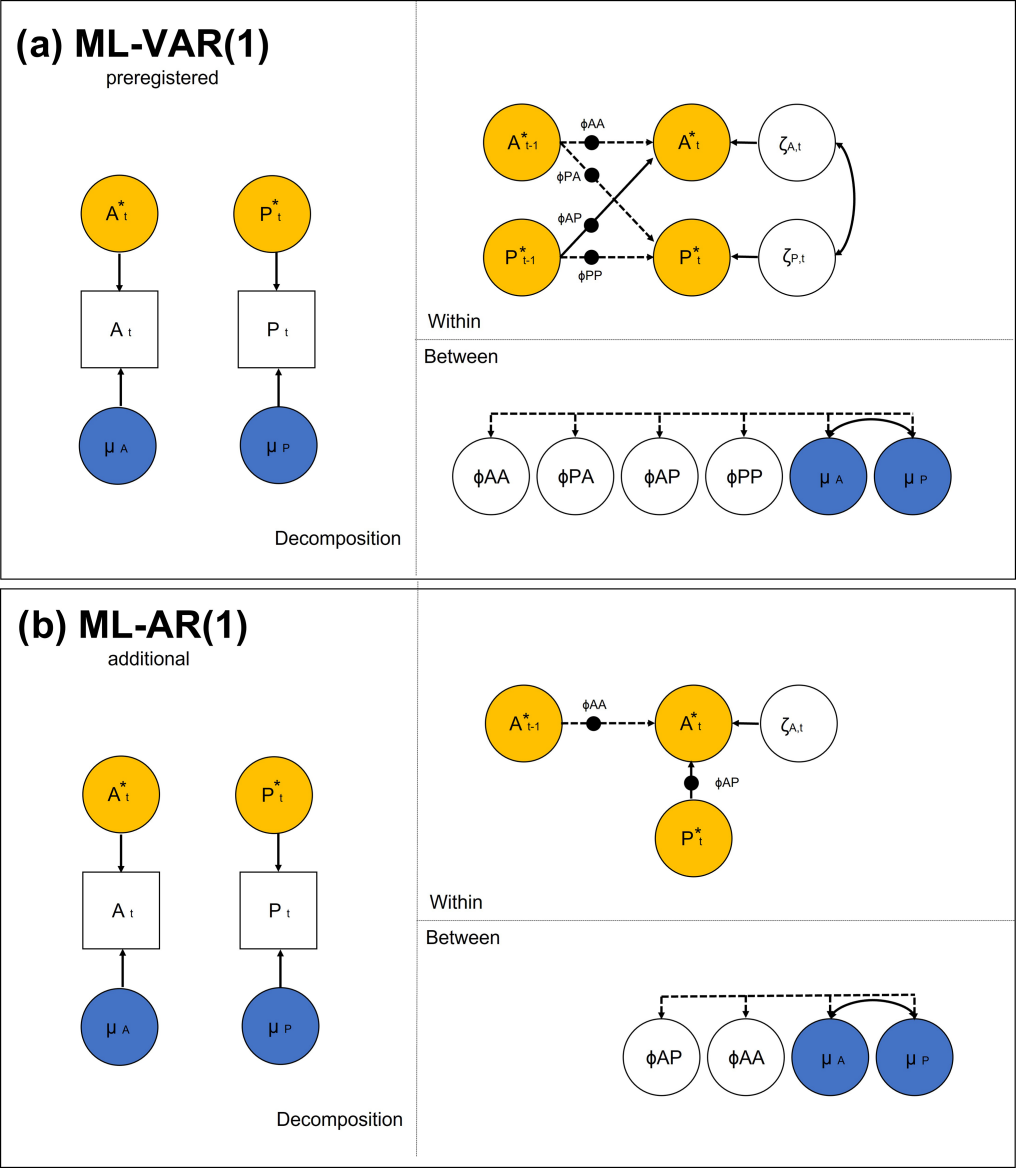
Specification of dynamic structural equation model. (a) represents specification of preregistered multilevel vector autoregressive models (ML‐VAR). (b) represents specifications of additional multilevel autoregressive models (ML‐AR). A = affect, P = parent‐adolescent interaction quality. Left: Variables are decomposed in between‐family part (μ = individuals mean), and within‐family part (A*_t_ = within‐person centered score of affect, P*_t_ = within‐person centered score of interaction quality). Top: On the within‐family level, affect and parent‐adolescent interaction quality predict each other over time (ϕ = autoregressive parameter, ζ = innovation). Solid arrows indicate hypothesized paths, dashed arrows are estimated without hypotheses. Bullets on lines indicate estimation of random effects (i.e., person‐specific effects). Bottom: On the between‐family level random effects and individual means are correlated. Figure is adapted from Hamaker and colleagues (Hamaker et al., 2018; McNeish & Hamaker, 2019).

As illustrated in Figure 1a, the reciprocal associations between interaction quality and adolescent affect were estimated on two levels, namely the within‐family and between‐family level. On the within‐family level, we specified the concurrent and lagged association between interaction quality and affect (H1). Using the “tinterval” option in M*plus*, the model accounts for unequal spacing between the measurements. As preregistered, the tinterval option was set to 3 h, as this is the mean time lag between ESM assessments. Therefore, the lagged associations can be interpreted as lags of 3 h. We furthermore specified the lagged associations as random effects, allowing to test for significant variance between families in person‐specific parameters (H3). On the between‐family level, we estimated the associations between the stable mean levels (i.e., random intercepts) of interaction quality and affect (H2), and the associations of these intercepts with random slopes of the cross‐lagged effects.

We checked convergence by inspecting the Gelman–Rubin statistics (i.e., potential scale reduction factors, PSR), density, and trace plots. If convergence was unsatisfactory, we followed our preregistered plan by increasing the number of iterations and increasing the thinning factor. In three of four models this led to a satisfactory model convergence. In one model (Conflict & Positive Affect), we detected a local convergence problem. By setting realistic start values for two parameters the model also reached a satisfactory convergence. We preregistered, that our hypotheses were confirmed if *p*‐values of unstandardized estimates were <.05 (two‐sided test for associations and one‐sided test for variance). Effect sizes were derived from standardized effect sizes (STDYX standardization in M*plus*; Muthén & Muthén, 2019; Schuurman et al., 2016). We further inspected differences between participants by exporting the person‐specific model results from the M*plus* output to R (Hallquist & Wiley, 2018; R Core Team, 2019). Sensitivity analysis show that our results are robust (for a detailed report see Supplemental Materials).

#### Deviations from the preregistration

The current paper deviates in three points from the preregistration: First, we also estimated a Multilevel Autoregressive Model (ML‐AR, see Figure 1b) to better conceptualize concurrent associations. This model allowed for a more direct test of concurrent effects (i.e., not controlling for previous levels of parenting; H1) and modeling heterogeneity in concurrent effects (H3). Second, we adapted our inference criteria. In DSEM Models credibility intervals should be preferred above *p*‐values (McNeish & Hamaker, 2019). Furthermore, both *p*‐values and credibility intervals cannot be used to test significance for variances, as the priors preclude negative values (McNeish & Hamaker, 2019). We, therefore, now use credibility intervals for H1 and H2 and a standard deviation effect ratio (Bolger et al., 2019) for H3 to infer significance. Third, we preregistered that participants would be removed by default who have no variance in their answers, however, this was not the default setting in M*plus*. Nine participants did not show variance on at least one scale (*n *= 7 warmth, *n *= 7 conflict, *n *= 4 positive affect, *n* = 5 negative affect). As these participants could still be included for estimating the between‐family estimates, we decided to include them in the analysis and run sensitivity tests without these participants afterwards (see sensitivity analysis). Please note, that all deviations of the preregistered plan were applied to test our hypotheses in the best statistical way and that they did not change any result.

## RESULTS

### Descriptive analysis

**FIGURE 2:**
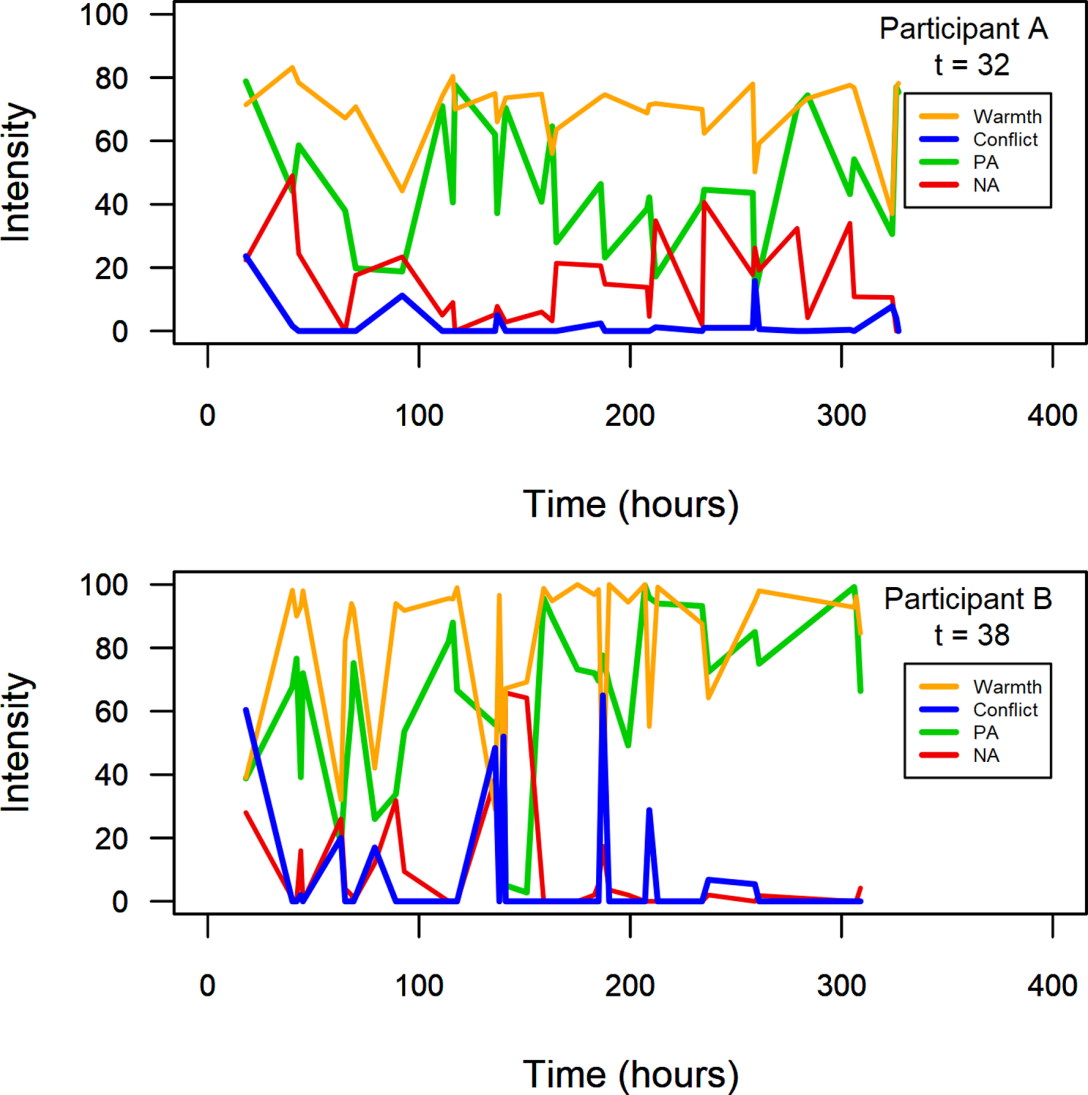
Fluctuations in experience sampling data for two participants. PA= positive affect, NA = negative affect

Table 1 displays the descriptive statistics and correlations of the study variables. As indicated by the Intraclass Correlation (ICC), a substantial proportion of the variance (up to 40%–72%) was due to moment‐to‐moment changes (i.e., 1 − ICC). Figure 2 illustrates such within‐family variance of two participants. All variables were correlated with each other, and the correlation pattern was in the same direction on the within‐ and between‐family level.

**TABLE 1:** Descriptive statistics and correlations for study variables

	*M*	*SD*	Min − Max	Skewness	Kurtosis	*ICC*	*ω_w_*	*ω_b_*	1	2	3	4
1. Warmth	75.81	19.58	0 − 100	−1.06	1.17	.61	.85	.97	—	−.55^**^	.41^**^	−.27^**^
2. Conflict	7.90	13.91	0 − 100	3.03	10.85	.28	.87	.95	−.50^**^	—	−.22^**^	.29^**^
3. PA	64.12	21.34	0 − 100	−0.51	−0.05	.60	.85	.95	.66^**^	−.19^*^	—	−.50^**^
4. NA	6.88	10.24	0 − 100	2.83	11.15	.35	.64	.84	−.35^**^	.52^**^	−.45^**^	—

Note

PA, positive affect; NA, negative affect; ICC, intraclass correlation coefficient; *ω_w_*, within‐family omega; *ω_b_*, between‐family omega. Between‐family correlations are presented under the diagonal, within‐family correlations are presented above the diagonal.

* *p *< .01.

** *p *< .001.

### Dynamic structural equation models

#### Within‐family associations (H1)

We hypothesized (H1) that over time fluctuations in interaction quality would be associated with adolescent affect within the same family. As predicted, concurrent within‐family associations were all significant (tested in preregistered ML‐VAR and additional ML‐AR models): Warmth was associated with more positive affect (ML‐VAR: *r* = .39; ML‐AR: *β* = .40) and with less negative affect (ML‐VAR: *r* = −.27; ML‐AR: *β* = −.27), and conflict was associated with less positive affect (ML‐VAR: *r *= −.22; ML‐AR: *β *= −.24) and with more negative affect (ML‐VAR: *r* = .28; ML‐AR: *β* = .25; see Tables 2 and 3).

**TABLE 2:** Model Results of Preregistered Dynamic Structure Equation Models (ML‐VAR) to test for lagged associations

	Positive affect					Negative affect				
	Est.	Est. St.	*p*	95% CI	95% HI	Est.	Est. St.	*p*	95% CI	95% HI
Within‐family (H1.1)										
Warmth (*t*) & Affect (*t*)	44.59	.39	**<.001**	**[37.26; 52.10]**	—	−18.36	−.27	**<.001**	**[−22.40; −14.43]**	—
Warmth (*t*) → Warmth (*t* + 1)	0.15	.16	.**008**	**[0.04; 0.25]**	[−0.58; 0.88]	0.19	.20	**<.001**	**[0.09; 0.30]**	[−0.64; 1.02]
Warmth (*t*) → Affect (*t* + 1)	0.09	.11	.158	[−0.03; 0.21]	[−0.89; 1.07]	−0.11	−.17	.**002**	**[−0.17; −0.04]**	[−0.63; 0.41]
Affect (*t*) → Warmth (*t* + 1)	−0.06	−.07	.316	[−0.18; 0.06]	[−1.02; 0.90]	0.03	.01	.834	[−0.21; 0.26]	[−1.68; 1.74]
Affect (*t*) → Affect (*t* + 1)	0.22	.20	**<.001**	**[0.11; 0.32]**	[−0.61; 1.05]	0.12	.11	.**026**	**[0.01;.022]**	[−0.66; 0.90]
Between‐family (H2.1)										
Warmth & Affect	223.76	.75	**<.001**	**[158.63; 317.22]**	—	−38.21	−.37	**<.001**	**[−67.15; −15.56]**	—
Variance (H3)										
Warmth (*t*) → Warmth (*t* + 1)	0.14	**2.49**	<.001	[0.09; 0.23]	—	0.18	**2.23**	<.001	[0.12; 0.26]	—
Warmth (*t*) → Affect (*t* + 1)	0.25	**5.56**	<.001	[0.16; 0.38]	—	0.07	**2.41**	<.001	[0.04; 0.11]	—
Affect (*t*) → Warmth (*t* + 1)	0.24	**8.16**	<.001	[0.15; 0.35]	—	0.76	**29.06**	<.001	[0.51; 1.15]	—
Affect (*t*) → Affect (*t *+ 1)	0.18	**1.93**	<.001	[0.13; 0.27]	—	0.16	**3.33**	<.001	[0.11; 0.24]	—
Within‐family (H1.2)										
Conflict (*t*) & Affect (*t*)	−22.11	−.22	**<.001**	**[−27.86; −16.51]**	**—**	16.06	.28	**<.001**	**[12.83; 19.41]**	—
Conflict (*t*) → Conflict (*t* + 1)	0.11	.12	.**014**	**[0.02; 0.20]**	[−0.51; 0.73]	0.11	.11	.**026**	**[0.01; 0.20]**	[−0.57; 0.79]
Conflict (*t*) → Affect (*t* + 1)	−0.06	−.06	.528	[−0.22; 0.12]	[−1.33; 1.21]	0.13	.15	.**002**	**[0.05; 0.22]**	[−0.52; 0.78]
Affect (*t*) → Conflict (*t* + 1)	0.15	.11	.**010**	**[0.04; 0.26]**	[−0.83; 1.13]	−0.06	−.02	.628	[−0.30; 0.18]	[−2.17; 2.05]
Affect (*t*) → Affect (*t* + 1)	0.27	.27	**<.001**	**[0.18; 0.35]**	[−0.38; 0.92]	0.12	.12	.**038**	**[0.01; 0.22]**	[−0.76; 1.00]
Between‐family (H2.2)										
Conflict & Affect	−37.67	−.30	.**008**	**[−71.51; −10.30]**	—	23.93	.72	**<.001**	**[15.48; 36.16]**	—
Variance (H3)										
Conflict (*t*) → Conflict (*t* + 1)	0.10	**2.87**	<.001	[0.05; 0.16]	—	0.12	**3.15**	<.001	[0.07; 0.19]	**—**
Conflict (*t*) → Affect (*t* + 1)	0.42	**10.80**	<.001	[0.28; 0.64]	—	0.11	**2.55**	<.001	[0.07; 0.17]	—
Affect (*t*) → Conflict (*t* + 1)	0.25	**3.33**	<.001	[0.17; 0.38]	—	1.16	**17.95**	<.001	[0.80; 1.71]	—
Affect (*t*) → Affect (*t* + 1)	0.11	**1.23**	<.001	[0.07; 0.16]	—	0.20	**3.73**	<.001	[0.14; 0.30]	—

Note

Est., unstandardized estimates; Est. St., Estimates for fixed within‐ and between‐family effects are standardized using the STDYX Standardization (Within‐Level Standardized Estimates Averaged over Clusters) in M*plus*, variances are standardized by this formula √Var/b. A value >0.25 is the criterium for a significant variance (Bolger et al., 2019); *p* = Bayesian equivalent to two‐sided *p*‐values. They are interpreted “as the proportion of the posterior distribution on the opposite side of 0 than the posterior mean” (McNeish & Hamaker, 2019) *p*‐values of variances are reported one‐sided. This was the preregistered inference criterium for the hypotheses, 95% CI = 95% Credibility interval of unstandardized values, interference criterium. 95% HI = 95% Heterogeneity interval indicates (under the assumption of normality distribution) that 95% of the person‐specific parameters in the population lie in this interval effect; b∓1.96√Var (Bolger et al., 2019; McNeish & Hamaker, 2019). Please note that priors in DSEM models preclude values to be negative, therefore CI and *p* values always indicate significance, and they have to be further inspected.

**TABLE 3:** Model Results of Additional Dynamic Structure Equation Models (ML‐AR) to test for concurrent associations

	Positive affect					Negative affect				
	Est.	Est. St.	*p*	95% CI	95% HI	Est.	Est. St.	*p*	95% CI	95% HI
Within‐family (H1.1)										
Warmth (*t*) → Affect (*t*)	0.44	.40	**<.001**	**[0.36; 0.51]**	[−0.11; 0.99]	−0.19	−.27	**<.001**	**[−0.23; −0.14]**	[−0.53; 0.15]
Affect (*t*) → Affect (*t *+ 1)	0.23	.23	**<.001**	**[0.13; 0.32]**	[−0.53; 0.99]	0.22	.23	**<.001**	**[0.13; 0.31]**	[−0.46; 0.90]
Between‐family (H2.1)										
Warmth & Affect	224.80	.75	**<.001**	**[159.59; 322.74]**	—	−35.07	−.39	**<.001**	**[−61.00; −15.03]**	—
Variance (H3)										
Warmth (*t*) → Affect (*t*)	0.08	**0.64**	<.001	[0.05; 0.13]	—	0.03	**0.91**	<.001	[0.02; 0.05]	—
Affect (*t*) → Affect (*t* + 1)	0.15	**1.68**	<.001	[0.11; 0.21]	—	0.12	**1.57**	<.001	[0.09; 0.18]	—
Within‐family (H1.2)										
Conflict (*t*) → Affect (*t*)	−0.28	−.24	**<.001**	**[−0.39; −0.19]**	[−0.83; 0.27]	0.25	.31	**<.001**	**[0.17; 0.32]**	[−0.30; 0.80]
Affect (*t*) → Affect (*t* + 1)	0.29	.29	**<.001**	**[0.19; 0.37]**	[−0.44; 1.02]	0.23	.24	**<.001**	**[0.14; 0.31]**	[−0.39; 0.85]
Between‐family (H2.2)										
Conflict & Affect	−32.04	−.24	.**032**	**[−65.02; −3.37]**	—	24.08	.61	**<.001**	**[14.78; 36.68]**	—
Variance (H3)										
Conflict (*t*) → Affect (*t*)	0.08	**1.01**	<.001	[0.03; 0.17]	—	0.08	**1.13**	<.001	[0.04; 0.13]	—
Affect (*t*) → Affect (*t* + 1)	0.14	**1.29**	<.001	[0.10; 0.20]	—	0.10	**1.37**	<.001	[0.07; 0.15]	—

Note

Est., unstandardized estimates; Est. St., Estimates for fixed within‐ and between‐family effects are standardized using the STDYX Standardization (Within‐Level Standardized Estimates Averaged over Clusters) in M*plus*, variances are standardized by this formula √Var/b. A value >0.25 is the criterium for a significant variance (Bolger et al., 2019); *p* = Bayesian equivalent to two‐sided *p*‐values. They are interpreted “as the proportion of the posterior distribution on the opposite side of 0 than the posterior mean” (McNeish & Hamaker, 2019) p values of variances are reported one‐sided. This is the preregistered inference criterium for the hypotheses, 95% CI = 95% Credibility interval of unstandardized values, interference criterium. 95% HI = 95% Heterogeneity interval indicates (under the assumption of normality distribution) that 95% of the person‐specific parameters in the population lie in this interval effect; b∓1.96√Var (Bolger et al., 2019; McNeish & Hamaker, 2019). Please note that priors in DSEM models preclude values to be negative, therefore CI and *p* values always indicate significance, and they have to be further inspected.

For lagged associations (tested in preregistered ML‐VAR models), as expected, warmth predicted less negative affect 3 h later (*β* = −.17), and conflict predicted more negative affect 3 h later (*β* = .15). However, contrary to our predictions, no significant lagged association was found from interaction quality to positive affect. We explored effects of adolescent affect on subsequent interaction quality. In three of four models, these associations were not significant. The only exception was positive affect, which predicted more conflict 3 h later (*β* = .11, see Table 2). In sum, H1.1 (within‐family association warmth and affect) and H1.2 (within‐family association conflict and affect) were supported in terms of concurrent associations (Tables 2 and 3) for both positive and negative affect. Regarding lagged associations (Table 2) only links with negative affect, but not with positive affect, were observed.

#### Between‐family associations (H2)

Tapping into group‐level correlations between estimated stable levels (i.e., intercepts) of interaction quality and affect, all between‐family associations were in line with our hypothesis (H2), both in the preregistered ML‐VAR models as well as in the additional ML‐AR models. In families with higher average levels of warmth, adolescents experienced more positive and less negative affect on average (ML‐VAR: PA: *r* = .75; NA: *r *= −.37; ML‐AR: PA: *r *= .75; NA: *r *= −.39), and a higher level of conflict compared to other families was associated with less positive and more negative affect (ML‐VAR: PA: *r* = −.30; NA: *r *= .72; ML‐AR: PA: *r *= −.24; NA: *r *= .61, see Tables 2 and 3). Therefore, H2.1 (between‐family associations between warmth and affect) and H2.2 (between‐family associations between conflict and affect) were confirmed.

#### Between‐family differences in within‐family associations (H3)

**FIGURE 3:**
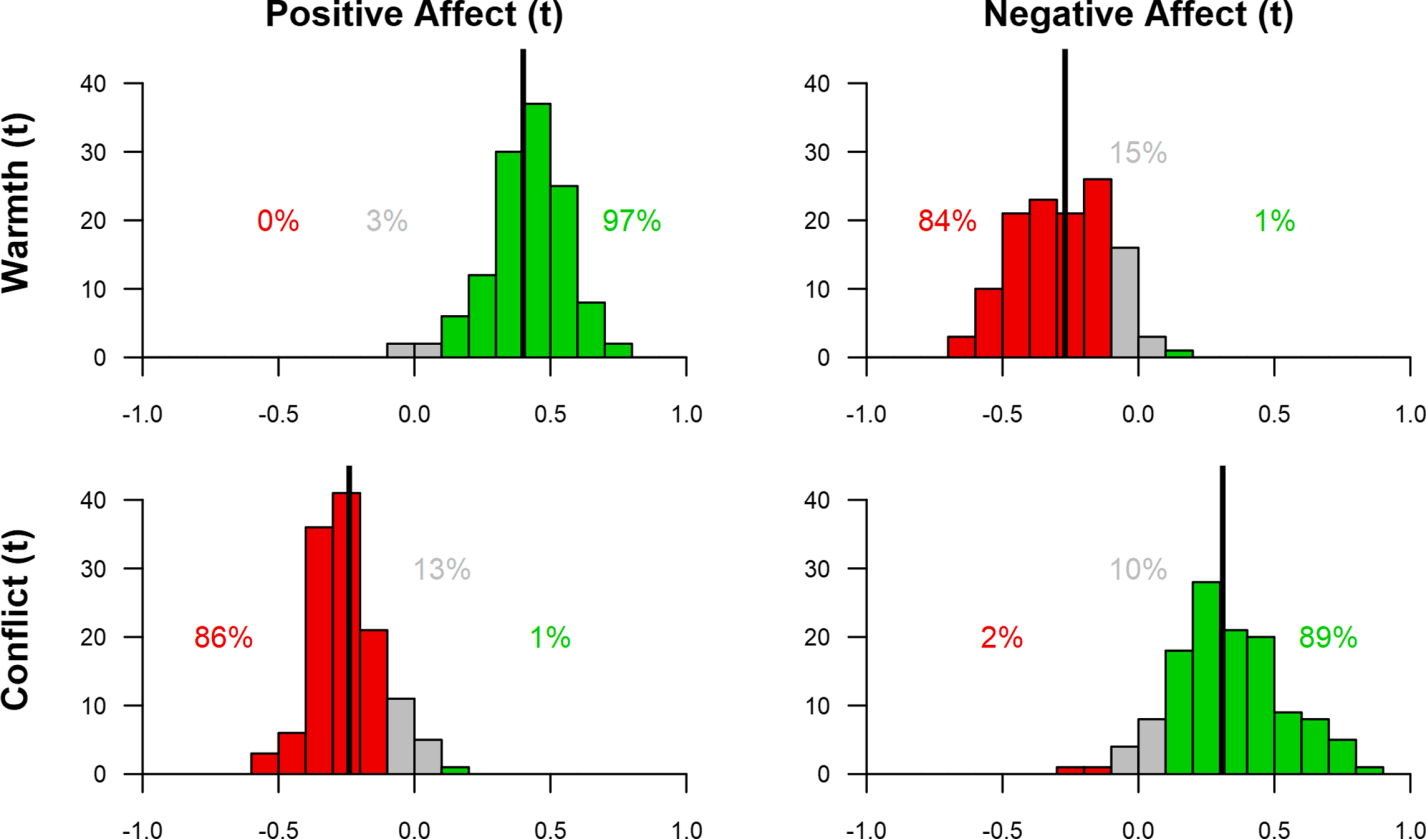
Distributions of person‐specific concurrent effects of interaction quality and affect. Percentages indicate the proportion of participants with a person‐specific standardized effect estimate of <‐.10 (red), between >‐.10 and <.10 (grey), and >.10 (green). This is a descrip‐tive summary and not based on statistical significance of these person‐specific parameters. Vertical line indicates average within‐person effect

**FIGURE 4:**
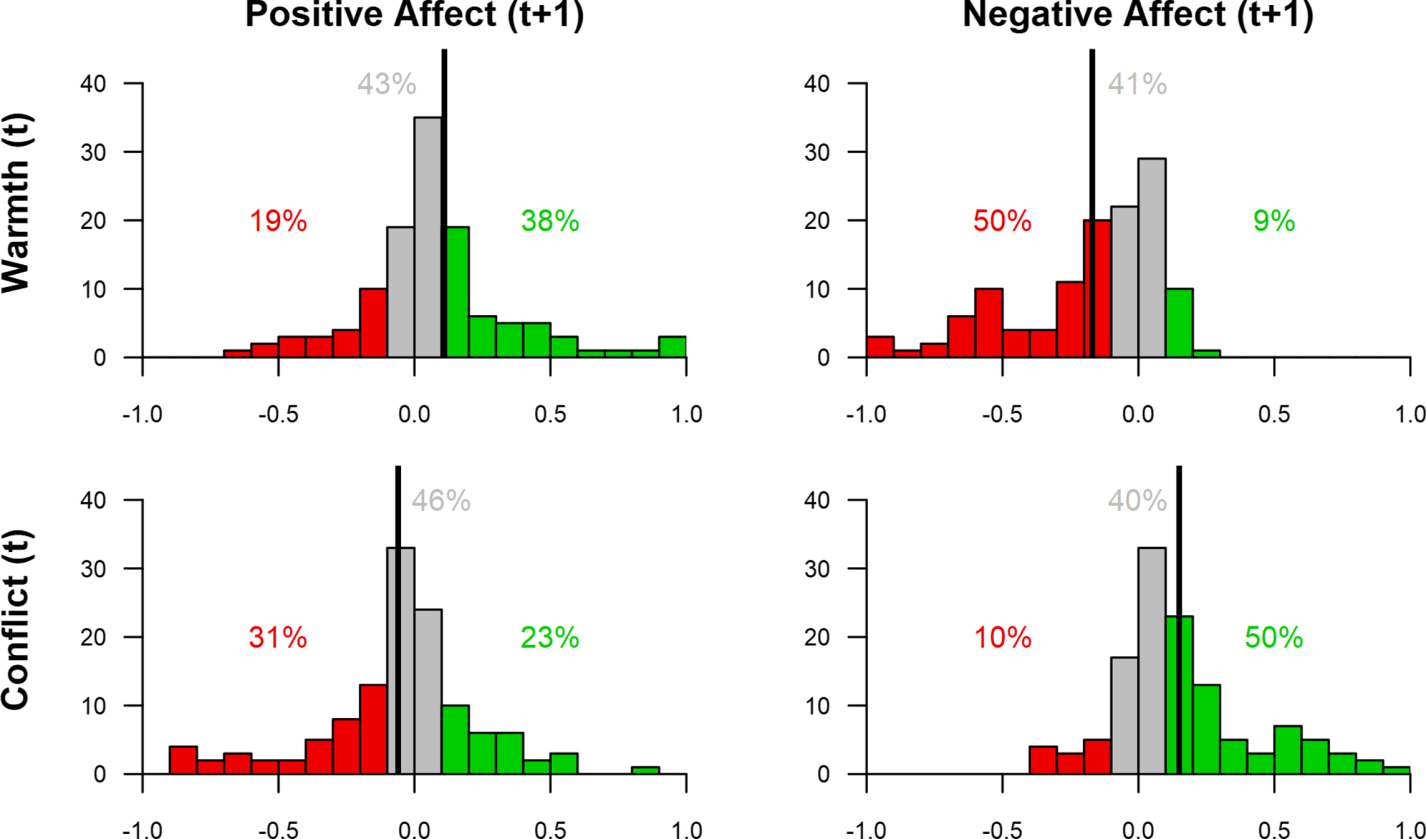
Distributions of person‐specific lagged effects from interaction quality to affect three hours later. Percentages indicate the proportion of participants with a person‐specific standardized effect estimate of <‐.10 (red), between >‐.10 and <.10 (grey), and >.10 (green). This is a descriptive summary and not based on statistical significance of these person‐specific parameters. Vertical line indicates average within‐person effect

Finally, we hypothesized effect heterogeneity, that is, we expected between‐family differences in within‐family concurrent and lagged effects (H3). Indeed, confirming our hypothesis, all concurrent and lagged within‐family associations had a significant variance around the estimated effects (all standardized variances >0.25, see Tables 2 and 3). Participants thus differed in the extent to which interaction quality was associated with affect on the same timepoint as well as over time. In Figures 3 and 4, the distribution of person‐specific parameters (i.e., the estimate per single family) are shown, indicating the differences between adolescents in their concurrent and lagged associations from interaction quality to affect. For the concurrent effects 36% and for the lagged effects, only 11% of these person‐specific parameters reached significance, probably due to insufficient power on the individual level. Therefore, person‐specific parameters should be cautiously interpreted on an individual level. However, the results show large differences between individuals. For concurrent effects, most participants had an effect in the expected direction (84%–97%), that is warmth co‐fluctuated with higher positive and lower negative affect, and conflict co‐fluctuated with lower positive and higher negative affect. In 3%–15%, there was no effect (−.10 < *β* < .10), while a few participants (0%–2%) showed opposite effects. For lagged effects the heterogeneity was even larger: in only 31%–50% of the participants the expected effect of interaction quality on subsequent affect was observed. While 40–46% showed no effect (−.10 < *β* < .10), and a substantial subgroup of participants (9%–23%) showed effects opposite to the hypothesized direction.

### Exploratory analysis: between‐family differences

We explored how the random slopes of our eight different models (2 [warmth/conflict] × 2 [positive/negative affect] × 2 [concurrent/lagged]) were correlated with each other (see Supplemental Materials). In brief, all concurrent effects were positively correlated with the cross‐lagged effects of the same model (*r* = .21–.59). That is, adolescents which, for example, experienced stronger increases in positive affect during a warmer interaction, compared to other adolescents, also experienced stronger increases of positive affect 3 h after a warm interaction. Furthermore, across models most concurrent associations were associated with each other (5 of 6 tests significant), indicating that adolescents who have stronger concurrent associations in one model (e.g., stronger increases in positive affect during a warm interaction) also have stronger associations in another model (e.g., stronger increases in negative affect during a conflict interaction). This pattern was also apparent in the cross‐lagged associations (5 of 6 tests significant). That is, for example, adolescents who showed stronger increases in positive affect after a warm interaction, compared to other adolescents, also showed stronger increases in negative affect after a conflict interaction.

We further explored if the strength of the within‐family associations depended on characteristics of adolescents, namely adolescent gender, age, and adolescent depressive symptoms. There were no differences between boys and girls in the effect of interaction quality to concurrent and subsequent affect (see Supplemental Materials). For age, two of eight tests were significant, namely, older adolescents, compared to younger adolescents, experienced a stronger increase in positive affect while having a warm interaction (*r* [*df* = 122] = −.19, *p *= .031), and stronger decrease in negative affect after having had a conflict (*r* [*df* = 122] = .13, *p *= .045). Furthermore, depressive symptoms were associated with effect heterogeneity in two of eight tests. Adolescents with more depressive symptoms, compared to adolescents with fewer depressive symptoms, showed stronger effects between interaction quality and subsequent affect: They experienced a stronger increase in positive affect (*r* [*df* = 116] = .19, *p *= .044) and stronger drop in negative affect (*r* [*df* = 116] = −.34, *p *< .001), after a warm interaction with their primary caregiver.

## DISCUSSION

Parent–child relationships are seen as one of the most proximal influences to child development (Bronfenbrenner, 1979; Sameroff, 2010). Even though much work has suggested that warm parent–adolescent relationships with few conflicts are associated with higher affective well‐being (meta‐analysis: Khaleque, 2013; Weymouth et al., 2016), this study is one of the first to assess these processes on a micro timescale employing ESM. Only very few studies have investigated the effects of presence of parents to adolescent affect with ESM (e.g., Kim et al., 2018; Offer, 2013) and to the best of our knowledge this is the first study to investigate the association of interaction quality and adolescent affect with ESM, while accounting for family‐specific processes.

Analyzing 2281 collected interactions of 124 adolescents, the study confirmed most of the preregistered hypotheses. At moments when parent–adolescent interactions were experienced as warmer, adolescents’ affect was also higher. Interactions that were experienced as more conflictual co‐occurred with decreased adolescent affect in daily life. For negative affect but not for positive affect, these effects of interaction quality on adolescent affect were still measurable 3 h later. As expected, there were substantial differences between families in these associations. In some families the interaction quality was strongly associated with adolescents’ affect, in other families the link was weak or non‐existent. When comparing families to each other (i.e., between‐family associations), adolescents who reported on average higher positive and lower negative affect than their peers experienced their interactions with parents as warmer and less conflictual.

### Micro process of parenting

Parent–adolescent relationships can be conceptualized as a stable phenomenon, but also as a dynamic process that fluctuates over time (Boele et al., 2020). Whereas much is known about how stable levels of interaction quality and adolescent affective well‐being are associated (meta‐analysis: Khaleque, 2013; Weymouth et al., 2016), very few studies have empirically tested fluctuations in daily life. In line with previous studies that observed moment‐to‐moment fluctuations in parenting (e.g., Hollenstein & Lewis, 2006) as well as day‐to‐day fluctuations (e.g., Bai et al., 2017), perceived parent–adolescent interaction quality varied from one interaction to the next. This variance was roughly as big as the variance between families (40%–72% within‐family variance). Such fluctuations around a stable equilibrium were meaningful, as deviations form how adolescents perceived their own parents could predict how they felt up to 3 h later. Such micro processes can possibly, bit by bit, alter long‐term developmental trajectories (Smith & Thelen, 2003).

However, our results suggest that the immediate effect of one interaction might be short‐lived and may vanish before the end of the day. Sensitivity analysis revealed that warmth significantly predicted negative affect 3 h but not 6 h later. This is in line with daily diary studies, which show inconsistent effects of interaction quality on next‐day adolescent affect (e.g., Boele et al., 2021; LoBraico et al., 2019; Timmons & Margolin, 2015). This highlights the added value of studying interaction quality at several moments within the day, by employing ESM to capture these micro processes. In sum our results are in line with our hypotheses and previous research, highlighting that (1) parent–adolescent interaction quality varies from moment to moment, (2) and predicts subsequent negative affect, however (3) this linkage might be short‐lived.

In line with transactional theory (Bell, 1968; Ramsey & Gentzler, 2015) suggesting that adolescents are active agents in influencing the interaction quality and therefore shaping their parent–adolescent relationship, the current study also explored the effect of affect on subsequent interaction quality. Adolescent positive affect predicted an increase in parent–adolescent conflict 3 h later. One could assume that adolescents with a heightened positive affect sometimes make decisions that parents do not approve of, due to a lower risk perception when experiencing more positive affect (Haase & Silbereisen, 2011). This could lead to a conflict a few hours later. This effect should be interpreted cautiously, however, as, (1) significant variance around this effect indicates that this effect did not apply to all participants, (2) the effect does not fully replicate in our sensitivity analysis, and (3) previous studies on adults did not find the same association between positive affect and conflict (Hawkley et al., 2007).

### Heterogeneity in associations between interaction quality and affect

Effect heterogeneity is a key feature of modern theories of parenting (e.g., Pluess & Belsky, 2010), and also the broader category of DST (e.g., De Ruiter et al., 2019), but was until recently hard to establish empirically. Our family‐specific approach detected substantial differences between families in their associations between interaction quality and affect, which is in line with prior daily‐diary research (e.g., Janssen et al., 2020). For example, the effect of warmth on adolescent positive affect 3 h later was ranging from strong positive (*β* = .97) to strong negative effects (*β* = −.63). Whereas, many families (43%) had no effect, 38% had the hypothesized positive association. However, 19% of our participants had a negative association. Such reverse effects challenge theoretical ideas stating that provision of warmth should be universally beneficial (Soenens et al., 2015, 2017). Even though this study was not specifically designed to draw inferences regarding individual families at the *n* = 1 level, our results reiterate the point that assuming universality (or heterogeneity) without testing it may easily lead to flawed conclusions (i.e., one size fits all fallacy; Bolger et al., 2019; Keijsers & Van Roekel, 2018). This calls for a new type of research in which not only the average effect is assessed in a sample, but also the variance between individuals in how they react to parenting. Assessing such family‐specific associations can answer fundamental theoretical questions, such as “Are there universal parenting principles?” (e.g., Soenens et al., 2015).

To get a first understanding of possible reasons why the association between interaction quality and affect differed between families, we also examined associations between different models and possible moderators. Adolescents who had stronger associations between warmth and affect also had stronger associations between conflict and affect. This possibly indicates that some adolescents are generally more susceptible toward parenting effects than others, both for better and for worse. Exploring possible moderators makes us cautiously conclude that older adolescents and adolescents with more depressive symptoms are the ones whose own affect is more susceptible for influences of the interaction quality with their parents. This is in line with earlier research on the daily level, which found that adolescents with more depressive symptoms were more susceptible to parental warmth and parent–adolescent conflict (Janssen et al., 2020; Timmons & Margolin, 2015), as well as differential susceptibility theory (Pluess & Belsky, 2010) stating that some children are more susceptible toward positive as well as negative environmental influences. Rather than universal mechanisms (Soenens et al., 2017), we conclude that the association between interaction quality with parents and adolescent affect differs substantially between families both in size and direction of effects. Adolescents’ depressive symptoms and age can potentially explain these differences.

### Limitations and future directions

Even though this study is among the first to assess how parent–adolescent interactions are dynamically linked with adolescent affect in daily life by analyzing 2281 interactions, there are also some notable limitations. Findings on one timescale cannot be readily applied to draw conclusions regarding another (Keijsers & Van Roekel, 2018). Conclusions from this study are therefore limited to the timescale under examination, and no inferences can be made on other timescales (e.g., longer term development). An important next step for future research is to investigate transactional linkage of parent–adolescent relationships and affective well‐being across different timescales by including multiple timescales (e.g., Boele et al., 2021) as well as their linkage in an integrative design (e.g., Borghuis et al., 2020). Such designs could investigate how the family‐specific micro processes could possibly manifest in different developmental trajectories.

Even though this study can significantly indicate heterogeneity in associations between families, our design was underpowered to reliably estimate person‐specific parameters. Therefore, results are not suited for interpreting individual parameters (i.e., idiographic approach/*N* = 1 approach; Molenaar et al., 2009), and should be cautiously interpreted on an individual level. Future research is needed to reliably estimate personalized parenting dynamics, by for example assessing more interactions per family (Neubauer et al., 2020). This could lead to a more in‐depth understanding of unique developmental trajectories and increase parenting interventions’ effectiveness by tailoring them to the individual characteristics of the family (Bamberger, 2016).

The study design limits the interpretation of findings in two ways. First because only adolescents reported on the adolescent–parent interactions, associations could be partly due to the subjective perception of the adolescent. Analyzing parents‐reports could possibly lead to different patterns (LoBraico et al., 2019). It is thus an open question to what extent interaction‐quality actually fluctuates between interactions and to what extent only adolescents’ perception changes from moment to moment. Therefore, future research should include parents’ perspective. Second, asking participants about the last interaction and their current affect, leads to a time lag even in measurements that we labeled as “concurrent.” That is, in our analysis of concurrent effects, interactions took place on average 14 min before adolescents rated their current affect.

Finally, we have to critically reflect on the generalizability of our results, as this study was based on a convenience sample and not all ESM questionnaires could be answered. Therefore, our study might be selective in the participants we included, but also in the moments that we measured. Even though the sample was quite diverse, including families with diverse ethnical and educational backgrounds, different family constellations and covers a wide age range of adolescent participants, we need replication studies to see if other samples with improved sampling designs show the same effects. Having an even more diverse sample could possibly lead to even greater differences in family dynamics. Our study, therefore, could possibly underestimate the variance of effects in the population.

## CONCLUSION

A DST approach toward parenting calls for novel methods to account for the complexity in developmental processes. By assessing parenting with ESM, this study explicitly modeled micro processes on the individual level that may differ between families. In almost all families, adolescent affect co‐fluctuated with the parent–adolescent interaction quality in terms of warmth and conflict. Moreover, parent–adolescent interaction quality predicted adolescents negative affect 3 h later. However, these lagged associations differed substantially between families in size and even direction of effects. These new insights into real‐time dynamics, may as such provide a first step to understanding family‐specific building blocks of development.

## CONFLICT OF INTERESTS

We have no known conflict of interest to disclose.

## Supporting information

Supplementary Material

## Appendix A

**TABLE A1:** Items

Construct	Original item (Dutch)	Translation
Warmth	Mijn ouder en ik konden goed overweg.	My parent and I got along well.
	Mijn ouder en ik hadden plezier.	My parent and I had fun.
	Mijn ouder gaf mij aandacht.	My parent gave me attention.
	Mijn ouder begreep mij.	My parent understood me.
	Mijn ouder luisterde naar mij.	My parent listened to me.
Conflict	Mijn ouder uitte kritiek op mij.	My parent criticized me.
	Mijn ouder ergerde zich aan mij.	My parent was annoyed by me.
	Mijn ouder was irritant.	My parent was annoying.
	Mijn ouder en ik waren het oneens.	My parent and I disagreed.
	Mijn ouder en ik waren aan het bekvechten.	My parent and I were quarreling.
Positive affect	Blij	Joyful
	Vrolijk	Cheerful
	Gelukkig	Happy
	Energiek	Lively
	Trots	Proud
Negative affect	Ellendig	Miserable
	Boos	Mad
	Angstig	Afraid
	Bang	Scared
	Verdrietig	Sad
Depressive symptoms	Ik voel me gelukkig*	I feel happy*
	Ik voel me eenzaam	I feel lonely
	Ik heb zin om me voor anderen te verstoppen	I like to hide from others
	Ik ben verdrietig	I am sad
	Ik heb zin om mezelf pijn te doen	I want to hurt myself
	Ik voel me een slecht mens	I feel like a bad person.
	Ik heb het gevoel dat ik niet deug	I feel like I am no good
	Ik ben kwaad over dingen	I am angry about things
	Ik verveel me	I am bored
	Ik heb het gevoel dat niets wat ik doe nog zin heft	I feel like nothing I do makes sense anymore

Instruction Warmth and Conflict: “Denk aan dat moment met je ouder wanneer je de volgende vragen gaat beantwoorden” [Think at the moment being with your parent while answering the following questions].

Instruction Affect: “Ik voel me nu …” [I now feel…], Instruction depressive symptoms: “Hieronder staan uitspraken die allemaal te maken hebben met bang zijn. Er zijn geen goede of foute antwoorden. Geef eerlijk aan hoe jij je meestal voelt.” [Below are statements that all have to do with being afraid. There are no right or wrong answers. Be honest about how you usually feel.]; * reverse coded (Table A1).

## Appendix B Deviation in procedure

The data from this study were composed from two substudies. The procedure of the pilot study (*N *= 30) deviated in three aspects from the main study (*N *= 99). Namely, the sampling scheme during the weekend, automatic reminders, the compensation for the study, the number of items per assessment, and the date the baseline questionnaire was sent (see Table B1). These changes were implemented to improve the study design.

**TABLE B1:** Differences in procedure in pilot and main study

	Pilot study	Main study
Sampling Scheme during Weekend	07:00–07:30	
		11:30–12:10
		13:30–14:10
	15:30–16:10	15:30–16:10
	17:30–18:10	17:30–18:10
	19:30–20:10	19:30–20:10
	21:30–22:00	21:30–22:00
Reminder	No reminder	Automatic reminder after 20 min
Compensation	5€ online questionnaire	5€ online questionnaire
		3€ compliance 65%–74%
	5€ compliance >74%	5€ compliance >74%
	Mood profile	Mood profile
	Invitation to guest lecture	Invitation to guest lecture
Number of items per assessment	25–32 items	34–37 items
Timing of baseline questionnaire	On day 1 (first day of ESM period)	On day 0 (one day before ESM period started)
