# Supplementary material for: Parent–adolescent interaction quality and adolescent affect—An experience sampling study on effect heterogeneity
Source: Child Dev. 2022 Jan 31;93(3):e315–31. doi: 10.1111/cdev.13733 (PMC9303236; doi:10.1111/cdev.13733)
Supplement: Supplementary file 1 — Supplementary Material [file CDEV-93-e315-s001.docx]

Supplemental Materials for

**Parent-adolescent interaction quality and adolescent affect - an experience sampling study on effect heterogeneity**

Anne Bülow^1,2^, Eeske van Roekel^2^, Savannah Boele^1,2^, Jaap Denissen^3^ & Loes Keijsers^1^

^1^ Department of Psychology, Education & Child Studies, Erasmus University Rotterdam,

^2^Department of Developmental Psychology, Tilburg University,

^3^ Department of Developmental Psychology, Utrecht University

**Author Note**

Anne Bülow <https://orcid.org/0000-0003-3335-7447>

Eeske van Roekel <https://orcid.org/0000-0002-0829-8089>

Savannah Boele <https://orcid.org/0000-0003-2821-1312>

Jaap Denissen <https://orcid.org/0000-0002-6282-4107>

Loes Keijsers <https://orcid.org/0000-0001-8580-6000>

**Open Practices.** The preregistered analytical plan (<https://osf.io/v6g2m/>) and codebook of the study (<https://osf.io/vstrn>) are shared on OSF.

**Disclosure of interests.** We have no known conflict of interest to disclose.

**Acknowledgements.** We are grateful for the participating families, and the support of Sari van Rooij, Claire Laudij-van Koot, and 41 students in collecting these data.

**Funding.** This research was supported by a grant from the Netherlands Organization for Scientific Research (NWO-VIDI; 452-17-011) awarded to Loes Keijsers.

**Correspondence.** Correspondence concerning this article should be addressed to Anne Bülow, Department of Psychology, Education and Child Studies, Erasmus University Rotterdam, P.O. Box 1738, 3000 DR Rotterdam, The Netherlands, Email: [bulow@essb.eur.nl](mailto:bulow@essb.eur.nl)

Table of Contents

[1 Psychometric properties of experience sampling scales 3](#_Toc86828828)

[1.1 Mplus Syntax for Multi-level Confirmatory Facotor Analysis 3](#_Toc86828829)

[1.2 Factor loadings per scale 6](#_Toc86828830)

[2 Power analysis 8](#_Toc86828831)

[2.1 Mplus Syntax for Monte Carlo simulations (Model 1) 9](#_Toc86828832)

[2.2 Mplus Syntax for Monte Carlo simulations (Model 2) 12](#_Toc86828833)

[2.3 Results of Monte Carlo Simulations 14](#_Toc86828834)

[3 Mplus Syntax for DSEM Analysis 16](#_Toc86828835)

[3.1 ML-VAR(1) models 16](#_Toc86828836)

[3.2 ML-AR(1) models 18](#_Toc86828837)

[4 Correlation between Random Slopes and Random Intercepts 20](#_Toc86828838)

[5 Sensitivity analysis 30](#_Toc86828839)

[6 Exploratory Analysis 36](#_Toc86828840)

[6.1 Associations between warmth and conflict over time. 36](#_Toc86828841)

[6.2 Between-Family Heterogeneity 37](#_Toc86828842)

[7 References 42](#_Toc86828843)

# 1 Psychometric properties of experience sampling scales

We estimated the factor loadings of all ESM scales using multi-level confirmatory factor analysis in M*plus* (Version 8.3, Muthén & Muthén, 2017). The Syntax is adapted from Geldhof and colleagues (2014).

### 1.1 Mplus Syntax for Multi-level Confirmatory Facotor Analysis

TITLE: CFA WARMTH

DATA:

FILE IS "DATA.dat";

VARIABLE:

NAMES are

ID hours NrMea

afESaa01 afESaa02 afESaa03 afESaa04 afESaa05

afESaa06 afESaa07 afESaa08 afESaa09 afESaa10

idESap01 idESap04

iqESap01 iqESap02 iqESap03 iqESap04 iqESap05

iqESap06 iqESap07 iqESap08 iqESap09 iqESap10

afESaaPA afESaaNA iqESapWA iqESapCO;

!name variables which you use in the analyses

USEVAR =

ID NrMea iqESap01 iqESap02 iqESap03 iqESap04 iqESap05;

!specify the time varying variable at within level

WITHIN = NrMea;

!cluster based on ID variable

CLUSTER = ID;

missing are all (5555 6666 7777 8888 9999);

ANALYSIS:

TYPE = TWOLEVEL;

PROCESSOR = 4;

MODEL:

%WITHIN%

OW BY iqESap01* (WL1)

iqESap02 (WL2)

iqESap03 (WL3)

iqESap04 (WL4)

iqESap05 (WL5);

OW@1;

iqESap01 (WR1);

iqESap02 (WR2);

iqESap03 (WR3);

iqESap04 (WR4);

iqESap05 (WR5);

%BETWEEN%

OB BY iqESap01* (BL1)

iqESap02 (BL2)

iqESap03 (BL3)

iqESap04 (BL4)

iqESap05 (BL5);

OB@1;

iqESap01 (BR1);

iqESap02 (BR2);

iqESap03 (BR3);

iqESap04 (BR4);

iqESap05 (BR5);

OUTPUT:

STANDARDIZED;

MODEL CONSTRAINT: NEW(NUMW DENOMW OMEGAW HW

NUMB DENOMB OMEGAB HB);

NUMW = (WL1+WL2+WL3+WL4+WL5)**2;

DENOMW = ((WL1+WL2+WL3+WL4+WL5)**2)+(WR1+WR2+WR3+WR4+WR5);

OMEGAW = NUMW/DENOMW;

HW = 1/(1+(1/((WL1**2/WR1)+(WL2**2/WR2)+(WL3**2/WR3)+(WL4**2/WR4)+(WL5**2/WR5))));

NUMB = (BL1+BL2+BL3+BL4+BL5)**2;

DENOMB = ((BL1+BL2+BL3+BL4+BL5)**2)+(BR1+BR2+BR3+BR4+BR5);

OMEGAB = NUMB/DENOMB;

HB = 1/(1+(1/((BL1**2/BR1)+(BL2**2/BR2)+(BL3**2/BR3)+(BL4**2/BR4)+(BL5**2/BR5))));

WR1 > 0; BR1 > 0;

WR2 > 0; BR2 > 0;

WR3 > 0; BR3 > 0;

### 1.2 Factor loadings per scale

**Table S1.1**

Results of Multilevel confirmatory factor model for parental warmth

|  | Factor loadings | |
| --- | --- | --- |
| Item | Within-family | Between-family |
| My parent and I got along well. | .80 | .94 |
| My parent and I had fun. | .60 | .77 |
| My parent gave me attention. | .69 | .97 |
| My parent understood me. | .80 | .99 |
| My parent listened to me. | .80 | .99 |

Note: Χ^2^ (df = 25) = 2363.63, p <. 001; CFI = .98; TLI = .96; RMSEA = .04

**Table S1.2**

Results of Multilevel confirmatory factor model for parent-adolescent conflict

|  | Factor loadings | |
| --- | --- | --- |
| Item | Within-family | Between-family |
| My parent criticized me. | .76 | .92 |
| My parent was annoyed by me. | .80 | .89 |
| My parent was annoying. | .76 | .95 |
| My parent and I disagreed. | .79 | .90 |
| My parent and I were quarreling. | .68 | .69 |

Note: Χ^2^ (df = 25) = 1299.64, p <. 001; CFI = .97; TLI = .97; RMSEA = .03

**Table S1.3**

Results of Multilevel confirmatory factor model for positive affect

|  | Factor loadings | |
| --- | --- | --- |
| Item | Within-family | Between-family |
| Joyful | .91 | .99 |
| Cheerful | .92 | .99 |
| Happy | .55 | .78 |
| Lively | .75 | .94 |
| Proud | .56 | .80 |

Note: Χ^2^ (df = 25) = 3209.16, p <. 001; CFI = .97; TLI = .95; RMSEA = .05

**Table S1.4**

Results of Multilevel confirmatory factor model for negative affect

|  | Factor loadings | |
| --- | --- | --- |
| Item | Within-family | Between-family |
| Miserable | .41 | .60 |
| Mad | .30 | .68 |
| Afraid | .85 | .97 |
| Scared | .86 | 1.00 |
| Sad | .45 | .65 |

Note: Χ^2^ (df = 25) = 866.14, p <. 001; CFI = .36; TLI = .07; RMSEA = .13

# 2 Power analysis

Prior to data collection of the main study (after collection of pilot study), a power analysis was conducted and preregistered ((<https://osf.io/v6g2m/>) using Monte Carlo simulations in M*plus* (Version 8.3, Muthén & Muthén, 2017) and R (Hallquist & Wiley, 2018; R Core Team, 2019) to determine the required sample size. Because we were primarily interested in within-person associations estimated with a bivariate multilevel vector autoregressive model (ML-VAR(1)), we investigated the power to detect these effects. We defined our smallest effect size of interest (SESOI) as β = .10, which is the equivalent of approximately 1% explained variance. Effects smaller than .10 were regarded as trivial. From our pilot study, we concluded that participants would roughly report on 20 parent-adolescent interactions per person.

Two types of Monte Carlo simulations were conducted. First, we simulated a bivariate multilevel vector autoregressive model, where the within-person cross-lagged effects were set to b = .10 (Model 1). Cross-lagged effects were allowed to vary among individuals (i.e., random effects were estimated). The results indicate that to estimate a small effect (b = .10) with 80% power, a sample of *N* = 70 with *t* = 20 would be needed (exact specifications see syntax below). Please note that this first approach was based on unstandardized effects and this would translate to an standardized effect of β = .13 and is therefore too liberal and not ideally suited for our sample size estimation.

Second, we simulated models based on realistic starting values derived from ML-VAR(1) models estimated in real data from an earlier independent pilot study in 49 Dutch adolescents (collected in 2018 with 952 mother-adolescent interactions). Our second approach (Model 2) led to a more cautious conclusion: to estimate a small effect of β = .08 with 80% power, a sample of *N* = 120 with *t* = 20 would be needed. In determining the sample size, we took the more conservative approach and strove for a sample size of *N* = 120 participants with ca. *t* = 20 observations per person to detect a SESOI of .10 (i.e., 2,400 observations). Ultimately, our sample of *N* = 124 participants yielded 2,281 reported interactions (95% of pre-registered sample size estimation).

### 2.1 Mplus Syntax for Monte Carlo simulations (Model 1)

Syntax was adapted from Masselink (2019)

Title: DSEM power calculations based on Maurits calculations;

Variable:

MONTECARLO: Names are W P ;

NOBSERVATIONS = 10000;

NCSIZES = 1;

CSIZES= 100 (100);

NREPS = 500;

SEED = 63498;

save = savedpower3.dat;

Within = ;

lagged = W P (1);

Analysis:

Type = twolevel random;

Estimator = Bayes;

biterations= 2001(2000);

PROCESSORS=2;

MODEL montecarlo:

%within%

!stability with lag1

ww| w ON w&1;

pp| p ON p&1;

!crosspaths with lag1

wp| w ON p&1;

pw| p ON w&1;

P*.70;

W*.70;

W with P*.10;

%between%

W with P*.10;

W*.76;

P*.76;

WW *0.010;

PP *0.010;

WP *0.010;

PW *0.010;

[WW *0.20];

[PP *0.20];

[WP *0.10];

[PW *0.10];

MODEL:

%within%

!stability with lag1

ww| w ON w&1;

pp| p ON p&1;

!crosspaths with lag1

wp| w ON p&1;

pw| p ON w&1;

P*.70;

W*.70;

W with P*.10;

%between%

W with P*.10;

W*.76;

P*.76;

WW *0.010;

PP *0.010;

WP *0.010;

PW *0.010;

[WW *0.20];

[PP *0.20];

[WP *0.10];

[PW *0.10];

OUTPUT: tech1 tech8 cinterval ;

### 2.2 Mplus Syntax for Monte Carlo simulations (Model 2)

TITLE: MONTE CARLO SIMULATION

MONTECARLO:

NAMES=warmth posaff;

lagged=warmth(1) posaff(1);

within = warmth posaff;

NREP = 100;

NOBSERVATIONS = 1600;

NCSIZES = 1;

CSIZES = 80 (20);! Participants(observations)

ANALYSIS:

TYPE IS TWOLEVEL random ;

ESTIMATOR=bayes;

PROC=4;

thin = 10;

MODEL MONTECARLO:

%WITHIN%

[posaff@0 warmth@0 ];

!stability with lag1

ww| warmth ON warmth&1 ;

pp| posaff ON posaff&1;

!crosspaths with lag1

wp| warmth ON posaff&1;

pw| posaff ON warmth&1;

! correlatie

warmth WITH posaff*0.480;

WARMTH * 1.955;

POSAFF * 1.625;

[WARMTH * 6.678];

[POSAFF * 4.856];

%between%

[ww * 0.254];

[pp * 0.073];

[wp * -0.432];

[pw * -0.040];

WW * 0.047;

PP * 0.046;

WP * 0.097;

PW * 0.040;

MODEL:

%WITHIN%

[posaff@0 warmth@0 ];

!stability with lag1

ww| warmth ON warmth&1 ;

pp| posaff ON posaff&1;

!crosspaths with lag1

wp| warmth ON posaff&1;

pw| posaff ON warmth&1;

! correlatie

warmth WITH posaff*0.480;

WARMTH * 1.955;

POSAFF * 1.625;

[WARMTH * 6.678];

[POSAFF * 4.856];

%between%

[ww * 0.254];

[pp * 0.073];

[wp * -0.432];

[pw * -0.040];

WW * 0.047;

PP * 0.046;

WP * 0.097;

PW * 0.040;

### 2.3 Results of Monte Carlo Simulations

**Table S2.1**

Power Estimates per Sample and Effect Size based on Monte Carlo Simulations of Model 1

| ***N*** | ***t*** | **A-B [.10]** | **BA [.10]** | **AB [.10]** | **Variance BA** | **Variance AB** |
| --- | --- | --- | --- | --- | --- | --- |
| 100 | 20 | 1.00 | 0.95 | 0.99 | 1.00 | 1.00 |
| 90 | 20 | 1.00 | 0.95 | 0.95 | 1.00 | 1.00 |
| 80 | 20 | 1.00 | 0.92 | 0.94 | 1.00 | 1.00 |
| 70 | 20 | 1.00 | 0.87 | 0.87 | 1.00 | 1.00 |
| 60 | 20 | 1.00 | 0.76 | 0.83 | 1.00 | 1.00 |
| 50 | 20 | 0.99 | 0.72 | 0.78 | 1.00 | 1.00 |
| 40 | 20 | 0.97 | 0.63 | 0.63 | 1.00 | 1.00 |
| 30 | 20 | 0.92 | 0.49 | 0.52 | 1.00 | 1.00 |

Note. 100 Simulations were estimated. N= number of participants, t = number of observations, A-B: concurrent within-family association between A and B. BA: within-family lagged association A (t) -> B (t+1), AB: within-family lagged association B (t) -> A (t+1). Size of effects are indicated in brackets. Green numbers are power >.80

**Table S2.2**

Power Estimates per Sample and Effect Size based on Monte Carlo Simulations of Model 2

| ***N*** | ***t*** | **P-W**  **[.27]** | **WP**  **[-.34]** | **PW**  **[-.04]** | **Variance**  **WP** | **Variance**  **PW** | **PP**  **[.08]** |
| --- | --- | --- | --- | --- | --- | --- | --- |
| 120 | 20 | 1.00 | 0.92 | 0.38 | 1.00 | 1.00 | 0.82 |
| 110 | 20 | 1.00 | 0.87 | 0.37 | 1.00 | 1.00 | 0.78 |
| 100 | 20 | 1.00 | 0.87 | 0.34 | 1.00 | 1.00 | 0.77 |
| 90 | 20 | 1.00 | 0.81 | 0.25 | 1.00 | 1.00 | 0.74 |
| 80 | 20 | 1.00 | 0.73 | 0.27 | 1.00 | 1.00 | 0.69 |
| 70 | 20 | 1.00 | 0.70 | 0.21 | 1.00 | 1.00 | 0.69 |
| 60 | 20 | 1.00 | 0.64 | 0.16 | 1.00 | 1.00 | 0.50 |
| 50 | 20 | 1.00 | 0.55 | 0.18 | 1.00 | 1.00 | 0.49 |
| 40 | 20 | 1.00 | 0.38 | 0.14 | 1.00 | 1.00 | 0.39 |
| 30 | 20 | 1.00 | 0.32 | 0.06 | 1.00 | 1.00 | 0.26 |

Note. 100 Simulations were estimated. N = number of participants, t = number of observations, P-W: concurrent within-family association between positive Affect and Warmth. WP: within-family lagged association Warmth (t) -> Positive affect (t+1), PW: within-family lagged association Warmth (t) -> Positive affect (t+1). PP: within-family lagged association Positive Affect (t) -> Positive affect (t+1) Size of effects are indicated in brackets. Green numbers are power >.80

# 3 Mplus Syntax for DSEM Analysis

### 3.1 ML-VAR(1) models

TITLE: DSEM ML-VAR(1): (Warmth & Pos) DATA

DATA: FILE = 'DATA.dat';

VARIABLE:

NAMES =

ID hours NrMea

afESaa01 afESaa02 afESaa03 afESaa04 afESaa05

afESaa06 afESaa07 afESaa08 afESaa09 afESaa10

idESap01 idESap04

iqESap01 iqESap02 iqESap03 iqESap04 iqESap05

iqESap06 iqESap07 iqESap08 iqESap09 iqESap10

afESaaPA afESaaNA iqESapWA iqESapCO; ! Names in Dataset

MISSING = ALL (5555 6666 7777 8888 9999); ! Missing Values

USEVAR = iqESapWA afESaaPA; !Variables in this analysis

! iqESapWA = Parental warmth

! afESaaPA = Positive Affect

CLUSTER = ID; ! Variable which identifies participants

LAGGED = iqESapWA(1) afESaaPA(1); ! create lagged variables

WITHIN = ;

TINTERVAL=hours(3);

!investiagated time interval 3 hours (initial settings)

ANALYSIS:

TYPE = TWOLEVEL RANDOM;

ESTIMATOR=BAYES;

BITER = 5000(100)!Initial settings max. 5 000 iterations min 100

PROC=2;

thin = 2; ! initial setting

MODEL:

! bivariate VAR(1) model with warmth and positive affect

%WITHIN%

!stability with lag1 (= 3 hours)

ww| iqESapWA ON iqESapWA&1;

pp| afESaaPA ON afESaaPA&1;

!hypothesized crosspaths with lag1 (= 3 hours)

!(H1: average effect + H3: random effect)

wp| iqESapWA ON afESaaPA&1;

pw| afESaaPA ON iqESapWA&1;

! H1: hypothesized within-person correlation between innovations

iqESapWA WITH afESaaPA;

%between%

!hypothesized between person correlations (H2)

!warmth with posaff;

!exploratory - and for better estimates

ww pp wp pw iqESapWA afESaaPA with ww pp wp pw iqESapWA afESaaPA;

output: TECH1 TECH8 TECH4(CLUSTER) standardized (cluster);

plot: type = plot3;

### 3.2 ML-AR(1) models

TITLE: DSEM ML-AR(1): (Warmth & Pos) DATA

DATA: FILE = 'DATA.dat';

VARIABLE:

NAMES =

ID hours NrMea

afESaa01 afESaa02 afESaa03 afESaa04 afESaa05

afESaa06 afESaa07 afESaa08 afESaa09 afESaa10

idESap01 idESap04

iqESap01 iqESap02 iqESap03 iqESap04 iqESap05

iqESap06 iqESap07 iqESap08 iqESap09 iqESap10

afESaaPA afESaaNA iqESapWA iqESapCO; ! Names in Dataset

MISSING = ALL (5555 6666 7777 8888 9999); ! Missing Values

USEVAR = iqESapWA afESaaPA; !Variables in this analysis

! iqESapWA = Parental warmth

! afESaaPA = Positive Affect

CLUSTER = ID; ! Variable which identifies participants

LAGGED = afESaaPA(1); ! create lagged variables

WITHIN = ;

TINTERVAL=hours(3);

!investiagated time interval 3 hours (initial settings)

ANALYSIS:

TYPE = TWOLEVEL RANDOM;

ESTIMATOR=BAYES;

BITER = 5000(100)!Initial settings max. 5 000 iterations min 100

PROC=2;

thin = 2; ! initial setting

MODEL:

! bivariate AR(1) model with positive affect and warmth as time varying co-variate

%WITHIN%

!stability with lag1 (= 3 hours)

pp| afESaaPA ON afESaaPA&1;

!hypothesized concurrent effect

!(H1: average effect + H3: random effect)

pw| afESaaPA ON iqESapWA;

! variance Positive Affect

afESaaPA;

! variance Warmth

iqESapWA;

%between%

%BETWEEN%

!Intercepts

[afESaaPA];

[iqESapWA];

[pp];

[pw];

!Variances

afESaaPA;

iqESapWA;

pp;

pw;

! correlation between parameters

afESaaPA iqESapWA pp pw WITH afESaaPA iqESapWA pp pw;

output: TECH1 TECH8 TECH4(CLUSTER) standardized (cluster);

plot: type = plot3;

# 4 Correlation between Random Slopes and Random Intercepts

| Variable | 1 | 2 | 3 | 4 | 5 | 6 |
| --- | --- | --- | --- | --- | --- | --- |
| 1 ΦWW | 1 |  |  |  |  |  |
| 2 ΦPP | -0.55** | 1 |  |  |  |  |
| 3 ΦWP | -0.35* | 0.55*** | 1 |  |  |  |
| 4 ΦPW | 0.48** | -0.70*** | -0.28* | 1 |  |  |
| 5 Warmth | -0.21 | 0.10 | 0.29 | -0.16 | 1 |  |
| 6 PA | -0.12 | -0.18 | 0.18 | -0.08 | 0.75*** | 1 |

In the multilevel vector autoregressive models (ML-VAR) random slopes and random intercepts were allowed to be correlated. These associations are reported in Table S4.1 – S4.4. For the multilevel autoregressive modela (ML-AR) these associations are reported in Table. S4.5 – S4.8. We had no hypotheses about these associations.

**Table S4.1**

Associations between random slopes and random intercepts for the Model Warmth and Positive Affect (ML-VAR)

Note: ΦWW = random slope of the stability path warmth (t) -> warmth (t+1), ΦPP = random slope of the stability path PA(t) -> PA(t+1), ΦWP = random slope of the cross-lagged path PA(t) -> warmth (t+1), ΦPW = random slope of the cross-lagged path warmth(t) -> PA (t+1), Warmth = random intercept of warmth, PA = random intercept of positive affect. * p <.05, ** p <.01, *** p <.001

**Table S4.2**

Associations between random slopes and random intercepts for the Model Warmth and Negative Affect (ML-VAR)

| Variable | 1 | 2 | 3 | 4 | 5 | 6 |
| --- | --- | --- | --- | --- | --- | --- |
| 1 ΦWW | 1 |  |  |  |  |  |
| 2 ΦNN | -0.39* | 1 |  |  |  |  |
| 3 ΦWN | 0.49** | -0.34* | 1 |  |  |  |
| 4 ΦNW | -0.29 | 0.55*** | -0.24 | 1 |  |  |
| 5 Warmth | -0.21 | -0.15 | -0.04 | 0.28 | 1 |  |
| 6 NA | 0.03 | -0.03 | 0.13 | -0.67*** | -0.37*** | 1 |

Note: ΦWW = random slope of the stability path warmth (t) -> warmth (t+1), ΦNN = random slope of the stability path NA(t) -> NA(t+1), ΦWN = random slope of the cross-lagged path NA(t) -> warmth (t+1), ΦNW = random slope of the cross-lagged path warmth(t) -> NA (t+1), Warmth = random intercept of warmth, NA = random intercept of negative affect. * p <.05, ** p <.01, *** p <.001

**Table S4.3**

Associations between random slopes and random intercepts for the Model Conflict and Positive Affect (ML-VAR)

| Variable | 1 | 2 | 3 | 4 | 5 | 6 |
| --- | --- | --- | --- | --- | --- | --- |
| 1 ΦCC | 1 |  |  |  |  |  |
| 2 ΦPP | 0.08 | 1 |  |  |  |  |
| 3 ΦCP | -0.16 | -0.53*** | 1 |  |  |  |
| 4 ΦPC | -0.11 | 0.26 | -0.08 | 1 |  |  |
| 5 Conflict | 0.38* | -0.11 | 0.58*** | 0.02 | 1 |  |
| 6 PA | -0.11 | -0.45** | -0.07 | 0.14 | -0.30** | 1 |

Note: ΦCC = random slope of the stability path conflict (t) -> conflict (t+1), ΦPP = random slope of the stability path PA(t) -> PA(t+1), ΦCP = random slope of the cross-lagged path PA(t) -> conflict (t+1), ΦPC = random slope of the cross-lagged path conflict(t) -> PA (t+1), Conflict = random intercept of conflict, PA = random intercept of positive affect. * p <.05, ** p <.01, *** p <.001

**Table S4.4**

Associations between random slopes and random intercepts for the Model Conflict and Negative Affect (ML-VAR)

| Variable | 1 | 2 | 3 | 4 | 5 | 6 |
| --- | --- | --- | --- | --- | --- | --- |
| 1 ΦCC | 1 |  |  |  |  |  |
| 2 ΦNN | -0.34* | 1 |  |  |  |  |
| 3 ΦCN | -0.21 | 0.43*** | 1 |  |  |  |
| 4 ΦNC | 0.16 | -0.56*** | -0.11 | 1 |  |  |
| 5 Conflict | 0.23 | 0.04 | -0.36 | -0.10 | 1 |  |
| 6 NA | 0.13 | 0.01 | -0.30* | 0.37* | 0.72*** | 1 |

Note: ΦCC = random slope of the stability path conflict (t) -> conflict (t+1), ΦNN= random slope of the stability path NA(t) -> NA(t+1), ΦCN = random slope of the cross-lagged path NA(t) -> conflict (t+1), ΦNC = random slope of the cross-lagged path conflict(t) -> NA (t+1), Conflict = random intercept of conflict, NA = random intercept of negative affect. * p <.05, ** p <.01, *** p <.001

**Table S4.5**

Associations between random slopes and random intercepts for the Model Warmth and Positive Affect (ML-AR)

| Variable | 1 | 2 | 3 | 4 |
| --- | --- | --- | --- | --- |
| 1 ΦPP | 1 |  |  |  |
| 2 ΦPW | -.32 | 1 |  |  |
| 3 Warmth | .04 | .09 | 1 |  |
| 4 PA | -.19 | -.17 | .75*** | 1 |

Note: ΦPP= random slope of the stability path PA(t) -> PA(t+1), ΦPW = random slope of the concurrent path warmth(t) -> PA (t), Warmth = random intercept of warmth, PA = random intercept of positive affect. * p <.05, ** p <.01, *** p <.001

**Table S4.6**

Associations between random slopes and random intercepts for the Model Warmth and Negative Affect (ML-AR)

| Variable | 1 | 2 | 3 | 4 |
| --- | --- | --- | --- | --- |
| 1 ΦNN | 1 |  |  |  |
| 2 ΦNW | -.36* | 1 |  |  |
| 3 Warmth | -.34** | .05 | 1 |  |
| 4 NA | .63*** | -.72*** | -.39*** | 1 |

Note: ΦNN= random slope of the stability path NA(t) -> NA(t+1), ΦNW = random slope of the concurrent path warmth(t) -> NA (t), Warmth = random intercept of warmth, NA = random intercept of negative affect. * p <.05, ** p <.01, *** p <.001

**Table S4.7**

Associations between random slopes and random intercepts for the Model Conflict and Positive Affect (ML-AR)

| Variable | 1 | 2 | 3 | 4 |
| --- | --- | --- | --- | --- |
| 1 ΦPP | 1 |  |  |  |
| 2 ΦPC | .31 | 1 |  |  |
| 3 Conflict | .06 | .17 | 1 |  |
| 4 PA | -.35** | .08 | -.24* | 1 |

Note: ΦPP= random slope of the stability path PA(t) -> PA(t+1), ΦPC = random slope of the concurrent path conflict(t) -> PA (t), Conflict = random intercept of conflict, PA = random intercept of positive affect. * p <.05, ** p <.01, *** p <.001

**Table S4.8**

Associations between random slopes and random intercepts for the Model Conflict and Negative Affect (ML-AR)

| Variable | 1 | 2 | 3 | 4 |
| --- | --- | --- | --- | --- |
| 1 ΦNN | 1 |  |  |  |
| 2 ΦNC | -.06 | 1 |  |  |
| 3 Conflict | .25 | -.01 | 1 |  |
| 4 NA | .59*** | .53*** | .61*** | 1 |

Note: ΦNN= random slope of the stability path NA(t) -> NA(t+1), ΦNC = random slope of the concurrent path conflict(t) -> NA (t), Conflict = random intercept of conflict, NA = random intercept of negative affect. * p <.05, ** p <.01, *** p <.001

**Table S4.9**

Associations between random slopes for all models

|  | 1 | 2 | 3 | 4 | 5 | 6 | 7 | 8 | 9 | 10 | 11 | 12 |
| --- | --- | --- | --- | --- | --- | --- | --- | --- | --- | --- | --- | --- |
| 1 WA(t)->PA (t) | 1 |  |  |  |  |  |  |  |  |  |  |  |
| 2 WA(t) ->PA (t+1) | **.26**** | 1 |  |  |  |  |  |  |  |  |  |  |
| 3 PA(t) -> WA (t+1) | **.24**** | **-.42***** | 1 |  |  |  |  |  |  |  |  |  |
| 4 WA (t) -> NA (t) | **-.55***** | -.16 | -.09 | 1 |  |  |  |  |  |  |  |  |
| 5 WA (t) -> NA (t+1) | **-.23**** | **-.43***** | .12 | **.59***** | 1 |  |  |  |  |  |  |  |
| 6 NA (t) -> WA (t+1) | .07 | .13 | **-.29**** | -.10 | **-.33***** | 1 |  |  |  |  |  |  |
| 7 CO (t) -> PA (t) | **-.36***** | -.09 | .02 | .17 | .00 | -.02 | 1 |  |  |  |  |  |
| 8 CO (t) -> PA (t+1) | **-.25**** | **-.24**** | .05 | .04 | .10 | .04 | **.21*** | 1 |  |  |  |  |
| 9 PA (t) -> CO (t+1) | -.10 | -.05 | **-.34***** | -.13 | -.07 | .01 | **.20*** | -.16 | 1 |  |  |  |
| 10 CO (t) -> NA (t) | **.54***** | .12 | .13 | **-.63***** | **-.38***** | .04 | **-.45***** | -.02 | -.12 | 1 |  |  |
| 11 CO (t) -> NA (t+1) | **.36***** | **.27**** | .00 | **-.36***** | **-.43***** | .09 | **-.19*** | **-.24**** | .08 | **.48***** | 1 |  |
| 12 NA (t) -> CO (t+1) | -.06 | -.02 | **.25**** | .15 | .09 | **-.22*** | -.03 | .06 | **-.37***** | -.06 | -.17 | 1 |

Note: WA = parental warmth, CO – Parent-adolescent conflict, PA = Positive Affect, NA = Negative Affect. * p <.05, ** p <.01, *** p <.001

**Figure S4.1**

Scatterplots of random slopes with random intercepts


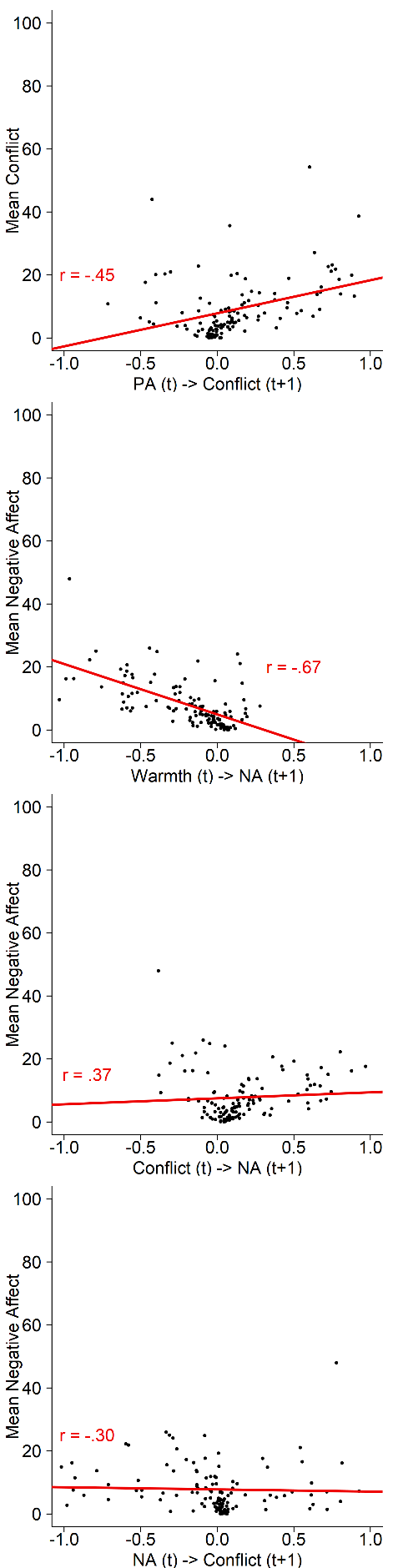


# 5 Sensitivity analysis

We preregistered several sensitivity analyses four our ML-VAR models to check the robustness of our findings. For each of the 4 models (2 (warmth/conflict) x 2 (positive/negative affect)), we estimated 6 additional models. First, we doubled the number of iterations to check for local minima. Second, we added the data of two participants who were identified as careless responders to see if their data would change the results. Third, we removed the data of participants with no within-person variance in the variables under investigation. Fourth, we estimated the models without a correlation between the random slopes. Fifth, we estimated with only fixed effects (i.e., no person-specific effects). And sixth, we set the time lag to 6 hours instead of 3 hours.

The results of these analyses are presented in Table S.5.1 to S.5.4. These indicated that the result pattern did not change when doubling the number of iterations, including data from 2 careless responders, or removing participants with no within-family variance. This suggests that the results are stable and not due to local minima or driven by specific participants. There was one exception: the explored effect of positive affect on subsequent conflict was not significant when removing participants without within-family variance. Results of models without random slopes or without correlation between random slopes affected mainly the cross-lagged effects. As our results indicate that there are substantial differences between participants in these effects, not accounting for these differences could have led to these changes in the result pattern. Increasing the time lag to six hours also reduced cross-lagged parameters, suggesting the here reported effects are possibly short-lived.

Furthermore, we also estimated Multilevel regression analysis in R to replicate our concurrent results in another statistical framework. Results are displayed in Table S.5.5. They, fully replicate the results from our DSEM analysis.

**Table S.5.1**

Sensitivity analysis Model 1 (Warmth & Positive Affect)

|  |  | Original Model | Double iterations | Number of participants | | Random specification | | Tinterval = 6 hrs |
| --- | --- | --- | --- | --- | --- | --- | --- | --- |
|  |  |  |  | + careless responders | - participants without variance | Without random slopes correlation | Without random slopes |  |
| ***Within-family (H1.1)*** |  |  |  |  |  |  |  |  |
| Warmth(t) & Affect (t) |  | **.39*** | **.38*** | **.38*** | **0.39*** | **.43*** | **.40*** | **.39*** |
| Warmth (t) -> Warmth (t+1) |  | **.16*** | **.15*** | **.15*** | **0.16*** | **.15*** | **.19*** | **.17*** |
| Warmth (t) -> Affect (t+1) |  | .11 | .10 | .10 | 0.10 | .06 | .03 | .04 |
| Affect (t) -> Warmth (t+1) |  | -.07 | -.05 | -.04 | -0.04 | -.03 | .06 | .02 |
| Affect (t) -> Affect (t+1) |  | **.20*** | **.21*** | **.22*** | **0.19*** | **.24*** | **.42*** | **.25*** |
|  |  |  |  |  |  |  |  |  |
| ***Between-family (H2.1)*** |  |  |  |  |  |  |  |  |
| Warmth & Affect |  | **.75*** | **.75*** | **.76*** | **0.75*** | **.77*** | **.72*** | **.76*** |
|  |  |  |  |  |  |  |  |  |
| ***Variance (H3)*** |  |  |  |  |  |  |  |  |
| Warmth (t) -> Warmth (t+1) |  | **2.49*** | **2.90*** | **2.44*** | **2.51*** | **1.98*** |  | **2.50*** |
| Warmth (t) -> Affect (t+1) |  | **5.56*** | **5.89*** | **6.14*** | **5.55*** | **5.76*** |  | **11.59*** |
| Affect (t) -> Warmth (t+1) |  | **8.16*** | **6.94*** | **8.45*** | **7.34*** | **9.27*** |  | **69.32*** |
| Affect (t) -> Affect (t+1) |  | **1.93*** | **2.02*** | **1.92*** | **2.11*** | **1.42*** |  | **1.48*** |

*Note*: Estimates are standardized using the STDYX Standardization (Within-Level Standardized Estimates Averaged over Clusters) Variances are standardized variances are standardized by this formula ${\surd Var}/b$(Bolger et al., 2019), * significant result, significance based on credibility intervals for H1 + H2, And standardized variances >0.25 is the criterium for H3.

**Table S.5.2**

Sensitivity analysis Model 2 (Warmth & Negative Affect)

|  |  | Original Model | Double iterations | Number of participants | | Random specification | | Tinterval = 6 hrs |
| --- | --- | --- | --- | --- | --- | --- | --- | --- |
|  |  |  |  | + careless responders | - participants without variance | Without random slopes correlation | Without random slopes |  |
| ***Within-family (H1.1)*** |  |  |  |  |  |  |  |  |
| Warmth(t) & Affect (t) |  | **-.27*** | **-.27*** | **-.27*** | **-0.27*** | **-.29*** | **-.28*** | **-.30*** |
| Warmth (t) -> Warmth (t+1) |  | **.20*** | **.21*** | **.20*** | **0.19*** | **.21*** | **.24*** | **.21*** |
| Warmth (t) -> Affect (t+1) |  | **-.17*** | **-.17*** | **-.18*** | **-0.14*** | **-.12*** | -.02 | -.07 |
| Affect (t) -> Warmth (t+1) |  | .01 | .01 | .02 | -0.00 | .03 | .03 | .07 |
| Affect (t) -> Affect (t+1) |  | **.11*** | **.12*** | **.12*** | **0.14*** | **.12*** | **.34*** | **.14*** |
|  |  |  |  |  |  |  |  |  |
| ***Between-family (H2.1)*** |  |  |  |  |  |  |  |  |
| Warmth & Affect |  | **-.37*** | **-.37*** | **-.38*** | **-0.37*** | **-.39*** | **-.40*** | **-.41*** |
|  |  |  |  |  |  |  |  |  |
| ***Variance (H3)*** |  |  |  |  |  |  |  |  |
| Warmth (t) -> Warmth (t+1) |  | **2.23*** | **2.13*** | **2.09*** | **2.16*** | **1.63*** |  | **1.78*** |
| Warmth (t) -> Affect (t+1) |  | **2.41*** | **2.46*** | **2.51*** | **3.13*** | **2.87*** |  | **3.53*** |
| Affect (t) -> Warmth (t+1) |  | **29.06*** | **38.08*** | **35.65*** | **96.93*** | **11.73*** |  | **4.08*** |
| Affect (t) -> Affect (t+1) |  | **3.33*** | **3.28*** | **2.96*** | **2.98*** | **2.92*** |  | **3.12*** |

*Note*: Estimates are standardized using the STDYX Standardization (Within-Level Standardized Estimates Averaged over Clusters) Variances are standardized variances are standardized by this formula ${\surd Var}/b$(Bolger et al., 2019), * significant result, significance based on credibility intervals for H1 + H2, And standardized variances >0.25 is the criterium for H3.

**Table S.5.3**

Sensitivity analysis Model 3 (Conflict & Positive Affect)

|  |  | Original Model | Double iterations | Number of participants | | Random specification | | Tinterval = 6 hrs |
| --- | --- | --- | --- | --- | --- | --- | --- | --- |
|  |  |  |  | + careless responders | - participants without variance | Without random slopes correlation | Without random slopes |  |
| ***Within-family (H1.2)*** |  |  |  |  |  |  |  |  |
| Conflict(t) & Affect (t) |  | **-.22*** | **-.22*** | **-.22*** | **-0.23*** |  | **-.22*** | **-.21*** |
| Conflict (t) -> Conflict (t+1) |  | **.12*** | **.11*** | **.12*** | **0.10*** |  | **.22*** | **.11*** |
| Conflict (t) -> Affect (t+1) |  | -.06 | -.07 | -.06 | -0.06 |  | -.04 | -.05 |
| Affect (t) -> Conflict (t+1) |  | **.11*** | **.13*** | **.11*** | 0.08 |  | .01 | .04 |
| Affect (t) -> Affect (t+1) |  | **.27*** | **.26*** | **.27*** | **0.27*** |  | **.42*** | **.27*** |
|  |  |  |  |  |  |  |  |  |
| ***Between-family (H2.2)*** |  |  |  |  |  |  |  |  |
| Conflict & Affect |  | **-.30*** | **-.30*** | **-.28*** | **-0.30*** |  | **-.20*** | **-.32*** |
|  |  |  |  |  |  |  |  |  |
| ***Variance (H3)*** |  |  |  |  |  |  |  |  |
| Conflict (t) -> Conflict (t+1) |  | **2.87*** | **2.85*** | **2.89*** | **2.60*** |  |  | **2.04*** |
| Conflict (t) -> Affect (t+1) |  | **10.80*** | **7.62*** | **6.67*** | **10.25*** |  |  | **6.65*** |
| Affect (t) -> Conflict (t+1) |  | **3.33*** | **3.30*** | **3.20*** | **4.76*** |  |  | **8.16*** |
| Affect (t) -> Affect (t+1) |  | **1.22*** | **1.24*** | **1.18*** | **1.15*** |  |  | **0.94*** |

*Note*: Estimates are standardized using the STDYX Standardization (Within-Level Standardized Estimates Averaged over Clusters) Variances are standardized variances are standardized by this formula ${\surd Var}/b$(Bolger et al., 2019), * significant result, significance based on credibility intervals for H1 + H2, And standardized variances >0.25 is the criterium for H3.

**Table S.5.4**

Sensitivity analysis Model 4 (Conflict & Negative Affect)

|  |  | Original Model | Double iterations | Number of participants | | Random specification | | Tinterval = 6 hrs |
| --- | --- | --- | --- | --- | --- | --- | --- | --- |
|  |  |  |  | + careless responders | - participants without variance | Without random slopes correlation | Without random slopes |  |
| ***Within-family (H1.2)*** |  |  |  |  |  |  |  |  |
| Conflict(t) & Affect (t) |  | **.28*** | **.28*** |  | **.28*** | **.31*** | **.28*** | **.27*** |
| Conflict (t) -> Conflict (t+1) |  | **.11*** | **.09*** |  | **.11*** | .06 | **.21*** | .09 |
| Conflict (t) -> Affect (t+1) |  | **.15*** | **.15*** |  | **.16*** | .00 | .05 | **.13*** |
| Affect (t) -> Conflict (t+1) |  | -.02 | -.02 |  | -.02 | .04 | .03 | .10 |
| Affect (t) -> Affect (t+1) |  | **.12*** | **.16*** |  | **.16*** | **.15*** | **.33*** | **.17*** |
|  |  |  |  |  |  |  |  |  |
| ***Between-family (H2.2)*** |  |  |  |  |  |  |  |  |
| Conflict & Affect |  | **.72*** | **.72*** |  | **.71*** | **.79*** | **.54*** | **.63*** |
|  |  |  |  |  |  |  |  |  |
| ***Variance (H3)*** |  |  |  |  |  |  |  |  |
| Conflict (t) -> Conflict (t+1) |  | **3.15*** | **3.32*** |  | **3.32*** | **4.07*** |  | **4.19*** |
| Conflict (t) -> Affect (t+1) |  | **2.55*** | **2.50*** |  | **2.39*** | **42.94*** |  | **3.63*** |
| Affect (t) -> Conflict (t+1) |  | **17.95*** | **25.07*** |  | **43.16*** | **46.48*** |  | **5.03*** |
| Affect (t) -> Affect (t+1) |  | **3.73*** | **3.77*** |  | **3.65*** | **2.33*** |  | **2.44*** |

*Note*: Estimates are standardized using the STDYX Standardization (Within-Level Standardized Estimates Averaged over Clusters) Variances are standardized variances are standardized by this formula ${\surd Var}/b$(Bolger et al., 2019), * significant result, significance based on credibility intervals for H1 + H2, And standardized variances >0.25 is the criterium for H3.

**Table S.5.5**

Multi-level regression

|  | Warmth -> PA | Warmth -> NA | Conflict -> PA | Conflict -> PA |
| --- | --- | --- | --- | --- |
| *Fixed effects:*  *Estimate (SE)* |  |  |  |  |
|  |  |  |  |  |
| Intercept | 9.17  (4.92) | 19.01***  (2.46) | 66.97***  (2.17) | 4.18***  (0.74) |
| BF Parenting -> Affect | 0.73***  (0.07) | -0.16***  (0.03) | -0.50**  (0.17) | 0.39***  (0.06) |
| WF Parenting-> Affect | 0.43***  (0.04) | -0.16***  (0.02) | -0.25***  (0.04) | 0.21***  (0.03) |
|  |  |  |  |  |
| *Random effects:* |  |  |  |  |
| WF Parenting -> Affect | 0.24 | 0.17 | 0.19 | 0.21 |
| Residual | 12.12 | 7.78 | 13.22 | 7.74 |

*Note*. BF = between-family effect, WF = within-family effect, Comparing the model with a model without random slope, always indicated significant better model fit for the model with random slope, indicating significant heterogeneity in this effect.

# 6 Exploratory Analysis

### 6.1 Associations between warmth and conflict over time.

To assess the interplay between different relationship dimensions, and to understand how warmth and conflict in one interaction affected warmth and conflict in future interactions, we furthermore explored the associations between warmth and conflict over time by conducting another ML-VAR(1) – Model. Results showed that on average more warmth was associated with more conflict in the same time point. Variances in all random effects were significant, indicating between-family differences in the patterning of parent-adolescent interactions over time.

**Table S.6.1**

Model results.

|  | Est. | Est. St. | *p^a^* | 95% CI |
| --- | --- | --- | --- | --- |
| ***Within-family*** |  |  |  |  |
| Warmth(t) & Conflict (t) | -54.07 | -.57 | **<.001** | **[-60.36; -48.23]** |
| Warmth (t) -> Warmth (t+1) | 0.09 | .09 | .426 | [-0.03; 0.21] |
| Warmth (t) -> Conflict (t+1) | 0.12 | .11 | .198 | [-0.01; 0.25] |
| Conflict (t) -> Warmth (t+1) | -0.12 | -.11 | .132 | [-0.28; 0.07] |
| Conflict (t) -> Conflict (t+1) | 0.18 | .19 | .540 | **[0.08; 0.27]** |
|  |  |  |  |  |
| ***Between-family*** |  |  |  |  |
| Warmth & Conflict | -66.86 | -.53 | **<.001** | **[-106.18; -38.14]** |
|  |  |  |  |  |
| ***Variance*** |  |  |  |  |
| Warmth (t) -> Warmth (t+1) | 0.29 | **5.98** | <.001 | [0.21; 0.42] |
| Warmth (t) -> Conflict (t+1) | 0.35 | **4.93** | <.001 | [0.25; 0.50] |
| Conflict (t) -> Warmth (t+1) | 0.28 | **4.41** | <.001 | [0.16; 0.47] |
| Conflict (t) -> Conflict (t+1) | 0.10 | **1.76** | <.001 | [0.06; 0.16] |

*Note*: Estimates are standardized using the STDYX Standardization (Within-Level Standardized Estimates Averaged over Clusters),

^a^ Bayesian equivalent to two-sided p-values, variances are reported one-sided as variances cannot be negative.

### 6.2 Between-Family Heterogeneity

As preregistered, we explored (i.e., without a priori hypotheses) if the cross-lagged associations between parenting and well-being would depend on the mean-level of either interaction quality and/or well-being (see Table S4.1 to S.4.4). In brief, adolescents with higher mean levels of negative affect, showed stronger decreases in negative effect after a warm interaction and stronger increases in negative affect after a conflictual interaction. In terms of reverse effects, from adolescent affect to conflict, adolescents with high levels of conflict showed stronger increase in conflictual interactions after experiencing more positive affect. Furthermore, adolescents with high mean levels of negative affect, showed stronger decreases in conflict after experiencing more negative affect. These exploratory findings show that parts of the effect heterogeneity can be explained by stable between-family differences in conflict and negative affect.

We explored how the random slopes of our eight different models (2 (warmth/conflict) x 2 (positive/negative affect) x 2 (concurrent/lagged)) were correlated with each other (see supplemental materials). In brief, all concurrent effects were positively correlated with the cross-lagged effects of the same model (*r* = .21 to .59). That is, adolescents which for example experienced stronger increases in positive affect during a warmer interaction, compared to other adolescents, also experienced stronger increases of positive affect three hours after a warm interaction. Furthermore, across models most concurrent associations were associated with each other (5 out of 6 tests significant), indicating that adolescents who have stronger concurrent associations in one model (e.g., stronger increases in positive affect during a warm interaction) also have stronger associations in another model (e.g., stronger increases in negative affect during a conflict interaction). This pattern was also apparent in the cross-lagged associations (5 out of 6 tests significant). That is, for example, adolescents who showed stronger increases in positive affect after a warm interaction, compared to other adolescents, also showed stronger increases in negative affect after a conflict interaction.

We further explored if the strength of the concurrent and cross-lagged associations depended on characteristics of adolescents, namely adolescent gender, age and adolescent depressive symptoms. There were no differences between boys and girls in the effect of interaction quality to subsequent affect (see Table S.6.2). For age, two out of eight tests were significant, namely, older adolescents, compared to younger adolescents, experienced a stronger increase in positive affect while having a warm interaction (*r* (*df* = 122) = -.19, *p* = .031), and stronger decrease in negative affect after having had a conflict (*r* (*df* = 122) = .13, *p* = .045; see Table S.6.3). Furthermore, as displayed in Figure S6.2, depressive symptoms were associated with effect heterogeneity. Adolescents with more depressive symptoms, compared to adolescents with fewer depressive symptoms, showed stronger effects between interaction quality and subsequent affect: They experienced a stronger increase in positive affect (*r* (*df* = 116) = .19, *p* = .044) and stronger drop in negative affect (*r* (*df* = 116) = -.34, *p* < .001; see Table S.6.4), after a warm interaction with their primary caregiver..

**Table S.6.2**

Gender differences

| *Variable* |  | *Girls* | |  | *Boys* | |  | *Difference* | | | |
| --- | --- | --- | --- | --- | --- | --- | --- | --- | --- | --- | --- |
|  |  | *M* | *SD* |  | *M* | *SD* |  | *t* | *df* | *p* | *d* |
| *Concurrent Associations* |  |  |  |  |  |  |  |  |  |  |  |
| Warmth (t)->PA (t) |  | 0.40 | 0.15 |  | 0.41 | 0.14 |  | 0.32 | 122 | .751 | 0.06 |
| Warmth (t)->NA (t) |  | -0.27 | 0.17 |  | -0.29 | 0.18 |  | 0.47 | 122 | .639 | 0.09 |
| Conflict (t)->PA (t) |  | -0.25 | 0.12 |  | -0.26 | 0.13 |  | 0.34 | 122 | .731 | 0.06 |
| Conflict (t)->NA (t) |  | 0.32 | 0.21 |  | 0.34 | 0.19 |  | 0.48 | 122 | .631 | 0.09 |
|  |  |  |  |  |  |  |  |  |  |  |  |
| *Lagged Associations* |  |  |  |  |  |  |  |  |  |  |  |
| Warmth (t)->PA (t+1) |  | 0.13 | 0.39 |  | 0.08 | 0.22 |  | 0.82 | 117.72^a^ | .416 | 0.14 |
| Warmth (t)->NA (t+1) |  | -0.19 | 0.30 |  | -0.16 | 0.26 |  | 0.75 | 122 | .456 | 0.14 |
| Conflict (t)->PA (t+1) |  | -0.06 | 0.31 |  | -0.04 | 0.27 |  | -0.28 | 122 | .776 | -0.05 |
| Conflict (t)->NA (t+1) |  | 0.17 | 0.29 |  | 0.14 | 0.23 |  | 0.50 | 122 | .621 | 0.09 |

^a^ correction for unequal variances

**Table S.6.3**

Association with Age

| *Variable* |  | *r* | *df* | *p* |
| --- | --- | --- | --- | --- |
| *Concurrent Associations* |  |  |  |  |
| Warmth (t)->PA (t) |  | -.19 | 122 | .031 |
| Warmth (t)->NA (t) |  | .11 | 122 | .244 |
| Conflict (t)->PA (t) |  | .02 | 122 | .866 |
| Conflict (t)->NA (t) |  | -.05 | 122 | .564 |
|  |  |  |  |  |
| *Lagged Associations* |  |  |  |  |
| Warmth (t)->PA (t+1) |  | .10 | 122 | .265 |
| Warmth (t)->NA (t+1) |  | -.14 | 122 | .113 |
| Conflict (t)->PA (t+1) |  | .11 | 122 | .210 |
| Conflict (t)->NA (t+1) |  | .13 | 122 | .045 |

**Figure S6.1**

Scatterplots of random slopes with age
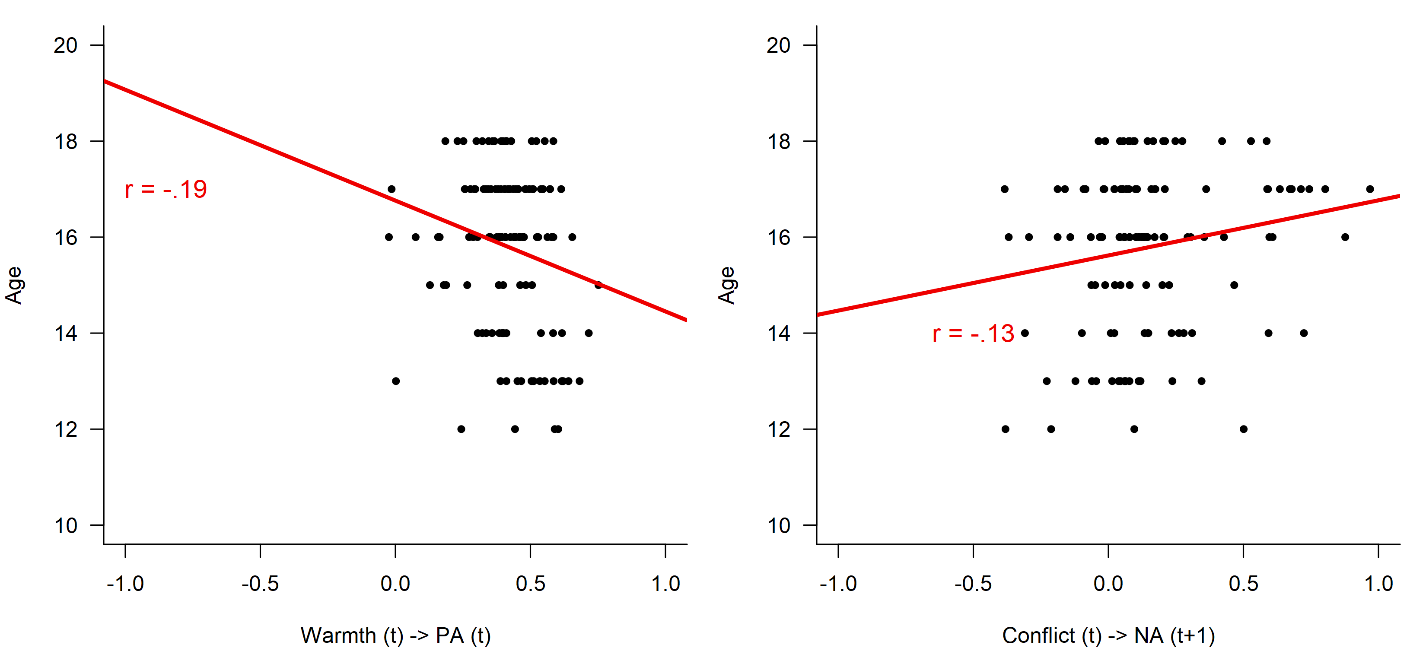


**Table S.6.4**

Association with Depressive Symptoms

| *Variable* |  | *r* | *df* | *p* |
| --- | --- | --- | --- | --- |
| *Concurrent Associations* |  |  |  |  |
| Warmth (t)->PA (t) |  | .03 | 116 | .764 |
| Warmth (t)->NA (t) |  | -.14 | 116 | .137 |
| Conflict (t)->PA (t) |  | .06 | 116 | .509 |
| Conflict (t)->NA (t) |  | .12 | 116 | .186 |
|  |  |  |  |  |
| *Lagged Associations* |  |  |  |  |
| Warmth (t)->PA (t+1) |  | .19 | 116 | .044 |
| Warmth (t)->NA (t+1) |  | -.34 | 116 | <.001 |
| Conflict (t)->PA (t+1) |  | -.04 | 116 | .681 |
| Conflict (t)->NA (t+1) |  | .12 | 116 | .219 |

**Figure S6.2**

Between-family Association between Adolescent Depressive Symptoms and Person-specific Associations between Interaction Quality and Affect


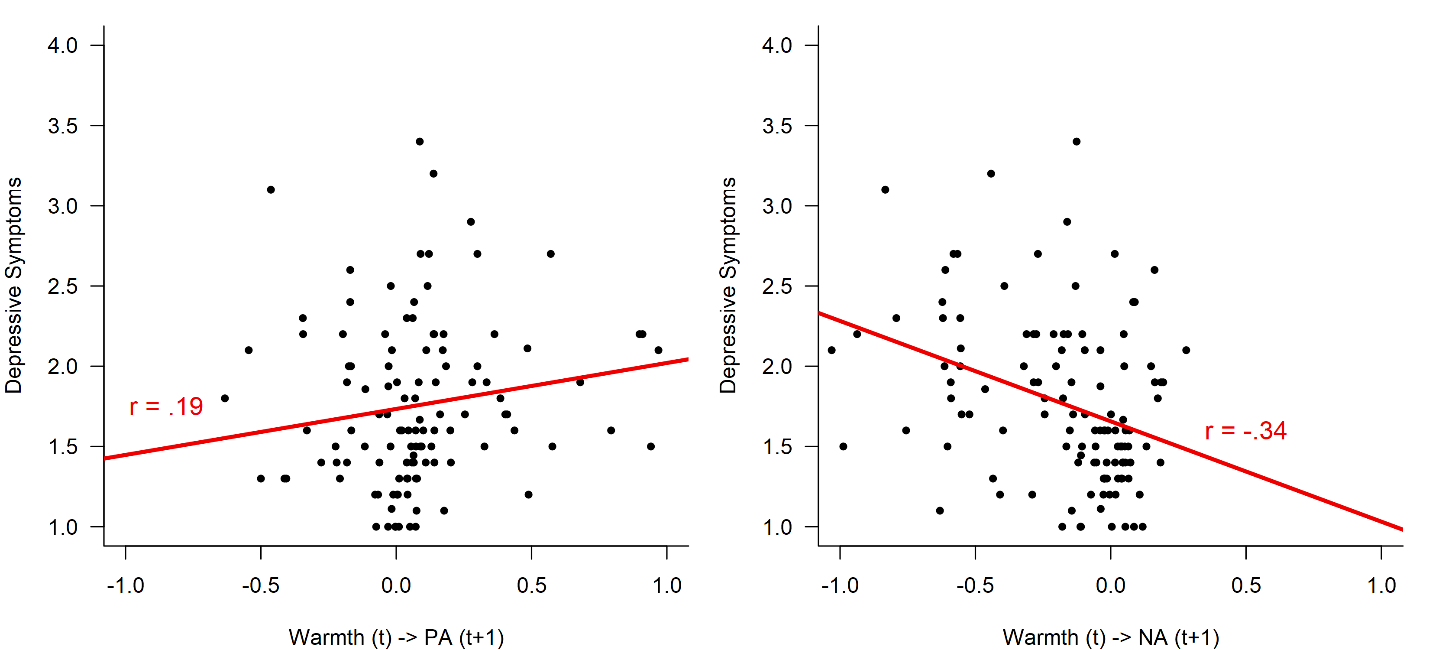


*Note*. PA= positive affect, NA = negative affect, t+1 = 3 hour interval

# 7 References

Geldhof, G. J., Preacher, K. J., & Zyphur, M. J. (2014). Reliability estimation in a multilevel confirmatory factor analysis framework. *Psychological Methods*, *19*(1), 72–91. https://doi.org/10.1037/a0032138

Hallquist, M. N., & Wiley, J. F. (2018). MplusAutomation: An R Package for Facilitating Large-Scale Latent Variable Analyses in Mplus. *Structural Equation Modeling*, *25*(4), 621–638. https://doi.org/10.1080/10705511.2017.1402334

Masselink, M. W. (2019). *Despicable me Self-esteem and depressive symptoms among adolescents and young adults*.

Muthén, L. K., & Muthén, B. (2017). *Mplus User’s Guide* (Muthén & Muthén (Eds.)).

R Core Team. (2019). *R: A language and environment for statistical computing. R Foundation for Statistical Computing*. https://www.r-project.org/
